# An integrated analysis of genes and pathways exhibiting metabolic differences between estrogen receptor positive breast cancer cells

**DOI:** 10.1186/1471-2407-7-181

**Published:** 2007-09-20

**Authors:** Soma Mandal, James R Davie

**Affiliations:** 1 Manitoba Institute of Cell Biology, University of Manitoba, 675 McDermot Avenue, Winnipeg Manitoba, R3E 0V9, Canada

## Abstract

**Background:**

The sex hormone estrogen (E2) is pivotal to normal mammary gland growth and differentiation and in breast carcinogenesis. In this *in silico *study, we examined metabolic differences between ER(+)ve breast cancer cells during E2 deprivation.

**Methods:**

Public repositories of SAGE and MA gene expression data generated from E2 deprived ER(+)ve breast cancer cell lines, MCF-7 and ZR75-1 were compared with normal breast tissue. We analyzed gene ontology (GO), enrichment, clustering, chromosome localization, and pathway profiles and performed multiple comparisons with cell lines and tumors with different ER status.

**Results:**

In all GO terms, biological process (BP), molecular function (MF), and cellular component (CC), MCF-7 had higher gene utilization than ZR75-1. Various analyses showed a down-regulated immune function, an up-regulated protein (ZR75-1) and glucose metabolism (MCF-7). A greater percentage of 77 common genes localized to the q arm of all chromosomes, but in ZR75-1 chromosomes 11, 16, and 19 harbored more overexpressed genes. Despite differences in gene utilization (electron transport, proteasome, glycolysis/gluconeogenesis) and expression (ribosome) in both cells, there was an overall similarity of ZR75-1 with ER(-)ve cell lines and ER(+)ve/ER(-)ve breast tumors.

**Conclusion:**

This study demonstrates integral metabolic differences may exist within the same cell subtype (luminal A) in representative ER(+)ve cell line models. Selectivity of gene and pathway usage for strategies such as energy requirement minimization, sugar utilization by ZR75-1 contrasted with MCF-7 cells, expressing genes whose protein products require ATP utilization. Such characteristics may impart aggressiveness to ZR75-1 and may be prognostic determinants of ER(+)ve breast tumors.

## Background

Breast cancer among other diseases, is a major cause of mortality in women, worldwide. Phenotypic changes during breast cancer progression reflect aberrant gene expression and pathways supporting deregulated growth. Thus, it is crucial to understand the events of initiation, transformation and metastasis using global gene expression approaches. Public database repositories of global gene expression data generated from high-throughput gene expression techniques such as SAGE and microarray (MA) can be successfully harnessed to gain meaningful insights to early detection, therapeutic outcome, patient assessment/survival, and drug development. Parallel to gene technology, the recently developed biocomputational tools help to understand the biology of a condition by the orderly arrangement of gene expression data.

The sex hormone E2 is pivotal to normal mammary gland growth and differentiation and its effects are directly related to the initiation and progression of breast cancer [1]. Targets of E2 associated signaling pathways comprise of several growth factors, growth factor receptors, extracellular proteins, immediate-early genes, and cell cycle regulators [2,3]. While many of these signaling molecules may contribute to E2 mediated mammary carcinogenesis, induction of their genes alone cannot fully explain the mitogenic effects of E2. Despite the identification of E2 targets by global gene expression studies, metabolic differences resulting from E2 deprivation of ER(+)ve breast cancer cells remain largely unexplored [4-6]. Pathways operating in ER(+)ve breast cancer cells in their un-induced state may be crucial determinants of downstream E2 effects and hence needs to be addressed. In this *in silico *study we used global gene expression data to perform biocomputational analysis to examine genes and pathways operating in E2 deprived luminal A type ER(+)ve breast cancer cell lines, MCF-7 and ZR75-1 [7].

## Methods

### Data processing and statistical analysis of SAGE libraries

**Figure 1:**
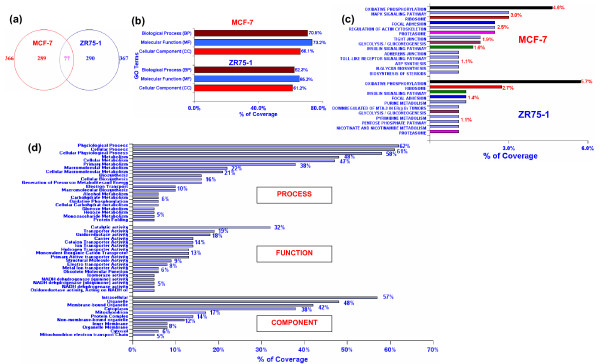
**Gene compartmentation and distribution in the GO terms and pathways**. **(a) **Venn diagram showing statistically significant genes (p ≤ 0.05, ≥5/≤-5) in MCF-7 and ZR75-1 compared to NBr. MCF-7 had 366 and ZR75-1 had 367 significant genes, and 77 genes intersecting between the circles were common. **(b) **In all the GO terms BP, MF, and CC, the gene distribution was higher for MCF-7 cells. Of all GO terms, function MF has the highest gene distribution in both cells. **(c) **DAVID selected 109 (30%) of 366 genes for MCF-7 cells, and 88 (24%) of 367 genes for ZR75-1 cells for KEGG's pathways, and the rest remained unclassified. The classified genes as shown are distributed in common pathways (oxidative phosphorylation, focal adhesion, insulin signaling, ribosome, proteasome, and glycolysis/gluconeogenesis). The steroid biosynthesis (1%), tight junction and Wnt signaling (0.8%, not shown) were predicted by DAVID for MCF-7. In ZR75-1 cells, highest gene utilization was seen in the oxidative phosphorylation pathway (5.7%), the rest were distributed in the ribosome (10 genes, 2.7%), the pentose phosphate (HMS), and proteasome pathways (1.1%) respectively. BIOCARTA pathway selected two other pathways with 35 (9.5%) genes, unselected by KEGG's pathway. Of these, 4, (1.1%) genes were involved in the MTA-3 in ER(-)ve breast tumor (more prominent in ZR75-1, GSEA), and 3 (0.8%) genes were involved in the glycolysis pathways. **(d) **GO classification of 77 common genes in MCF-7 and ZR75-1. Percent of coverage represents the percent of genes annotated by DAVID to the GO terms, BP, MF, and CC. Unclassified genes are not shown.

Public repositories of gene expression data obtained from SAGE and MA were used in this study [8,9]. SAGE libraries were generated from MCF-7 and ZR75-1 cells cultured in phenol red free medium with charcoal stripped FBS; these cells represented the 0 h time point (un-induced) of a E2 exposure time course experiment [4,10]. Breast cancer cells were compared with the NBr library generated from normal mammary cells purified from reduction mammoplasty tissue [11]. Raw sequences from SAGE libraries were analyzed by the SAGE software 2000 (V4.5) and extracted tags were compared between NBr and MCF-7 (NBr/MCF-7) and ZR75-1 (NBr/ZR75-1) (Table 1 lists the SAGE libraries used) [12]. Due to the non-availability of raw sequences of ZR75-1, data for this library were downloaded from NCBI [8]. We used Audic-Claverie, Fisher and Chi square statistical tests (IDEG6 software) to compare libraries [13]. Data files were annotated with the reference library (MS Access), further verified with SAGEMap tool for library annotation, and also compared with Absolute Level Lister (SAGEMap). Using MS Access, we created five files from two parent files (NBr/MCF-7 and NBr/ZR75-1). These files were NBr/MCF-7 (366 genes), NBr/ZR75-1 (367 genes), 77 common genes (MCF-7 and ZR75-1), 289 genes specific to NBr/MCF-7, and 290 genes specific to NBr/ZR75-1 respectively (Figure 1a). Regression analysis was performed to show the relationship between common genes; chromosomal localization of common genes was done using MAPviewer [8]. In addition, 263 differentially expressed genes identified within the SAGE libraries (advanced query function, MS Access) were arranged in a cluster by Tree View [14]. Genes whose expression satisfied the significance (p ≤ 0.05) and fold change criteria (≥5/≤-5, MCF-7/NBr or NBr/MCF-7 and ZR75-1/NBr or NBr/ZR75-1) were used. To normalize for interlibrary count differences, tag counts were converted to tags per million (TPM) for fold change calculations.

**Table 1:** A tabular summary of the publicly available SAGE libraries used for comparative analysis in this study

**SAGE Libraries Used**	**No. of Total tags**	**No. of Unique tags**
SAGE Breast normal epithelium AP NBr	50512	19190
SAGE Breast carcinoma CL ZR75-1 untreated	32303	3983
SAGE Breast carcinoma CL MCF-7 control 0 h	59877	15401
SAGE_Breast_carcinoma_CL_MDA435C	47270	20080

**Total tags**	**189962**	**58654**

MCF-7 and ZR75-1 cells were compared with NBr. A second comparison was made between MCF-7, ZR75-1, and MDA-MB-435 cells.

To substantiate our findings in the cell lines, we did multiple comparisons using the 263 gene dataset. Comparison with a basal-like ER(-)ve cell line MDA-MB-435 yielded 215 consensus genes, which were used for cluster analysis. Comparison between MCF-7 (SAGE) and cycling MCF-7 cells (MCF-7 NCI, cell isolate from NCI60 cell line set; MA) yielded 115 consensus genes, which were compared by regression analysis. Due to unavailability of expression data in SAGE/MA databases for cycling ZR75-1 cells, we could not do such comparison. Finally, we also compared cell lines and breast tumors (subtypes, ER(+)ve luminal A and B, ER(-)ve basal tumors, MA database) using gene set enrichment analysis (GSEA, V2.0) tool. Of the 228 genes selected by this tool, 163 genes were consensus. In all analyses, the MS Access advanced query function and manual inspection were used to remove duplicate genes. These files were used to create new files with specific file format for each bioinformatics tool.

### GO annotation, Pathway analysis and Gene Set Enrichment Analysis (GSEA)

Gene classification based on GO terms identifies gene families regulating a particular biological process (BP), molecular function (MF), and cellular component (CC). DAVID (V2.1), a multifunctional tool, annotates genes by functional classification and biochemical pathways [15]. Genes were annotated individually, for the common genes, and also for distinct clusters of differentially expressed genes. The pathway visualization tool, GenMAPP (V2.0) was used to visualize genes within pathways [16,17]. Up/down-regulated genes expressed in MCF-7/ZR75-1 were excluded from GenMAPP analysis unless they were above or below the fold change cutoff (with respect to NBr). To correlate the findings in cell lines with breast tumors, we used the gene enrichment tool, GSEA for five independently derived gene datasets (cell lines, tissues) [18].

## Results

### Gene utilization in the Gene Ontology (GO) terms and pathways and chromosomal gene localization in MCF-7 and ZR75-1 cells

ER(+)ve MCF-7 and ZR75-1 cells grown under E2 depleted conditions represent the un-induced state [4,10]. SAGE libraries from breast cancer cells were compared to the NBr library (normal breast) and statistically significant genes were chosen by fold change and significance criteria [11]. GO analysis was performed at three levels. Genes expressed in MCF-7 and ZR75-1 cells, common genes (77 genes), and genes forming distinct cluster signatures (CS) were annotated to delineate individual, common, and collective similarities and differences with NBr.

Individual annotation of 355 genes (of 366, MCF-7) and 355 genes (of 367, ZR75-1) by DAVID showed that the percent of classified gene utilization within the GO terms, BP, MF, and CC was higher in MCF-7 relative to ZR75-1 cells, although there was a similarity in gene distribution pattern (Figure 1b, Table 2). For the known pathways, KEGG's and BIOCARTA, DAVID annotated 109 (30%) of 366 (MCF-7) and 88 (24%) of 367 genes (ZR75-1), which were distributed in various pathways (Figure 1c). Common utilized pathways included the ribosome, proteasome, insulin signaling, oxidative phosphorylation/electron transport, and glycolysis/gluconeogenesis, while specific pathways utilized by MCF-7 included MAPK, tight junction, Toll-like receptor signaling, and steroid biosynthesis respectively. ZR75-1 cells also used the pentose phosphate (HMS) and MTA-3 pathways (Tables 1a,1b, see additional file 1). The individual highest gene distribution was seen in the oxidative phosphorylation/electron transport but not in glycolysis/gluconeogenesis pathways (Figure 1c). Within the common genes however, there was decreased similarity in GO terms related to metabolism (for BP, glucose carbohydrate, glucose/hexose metabolism) and energy (oxidative phosphorylation, BP, NADH dehydrogenase and reductase activity, for MF, and mitochondrial electron transport chain, CC) (Figure 1d; see additional file 1, Table 1c).

**Table 2:** Gene distribution in the individual GO terms

	**MCF-7**	**No. of Genes**	**%**	**ZR75-1**	**No. of Genes**	**%**
Process (BP)	Physiological Process	238	65.0	Cellular Process	214	58.5
	Cellular Process	232	63.4	Physiological Process	210	57.4
	Cellular Physiological Process	217	59.3	Cellular Physiological Process	202	55.2
	Metabolism	179	48.9	Metabolism	156	42.6
Function (MF)	Catalytic Activity	109	29.8	Catalytic Activity	93	25.4
	Protein Binding	55	15.0	Protein Binding	47	12.8
	Hydrolase Activity	48	13.1	Transporter Activity	40	10.9
	Transporter Activity	39	10.7	Hydrolase Activity	29	7.9
Component (CC)	Cell	229	62.6	Cell	212	57.9
	Intracellular	199	54.4	Intracellular	173	47.3
	Organelle	174	47.5	Organelle	155	42.3
	Membrane-bound Organelle	143	39.1	Membrane-bound Organelle	131	35.8

The distribution of genes within the individual GO terms was higher in MCF-7 cells compared to ZR75-1, though the pattern of gene distribution was similar. % denotes percentage of coverage.

**Figure 2:**
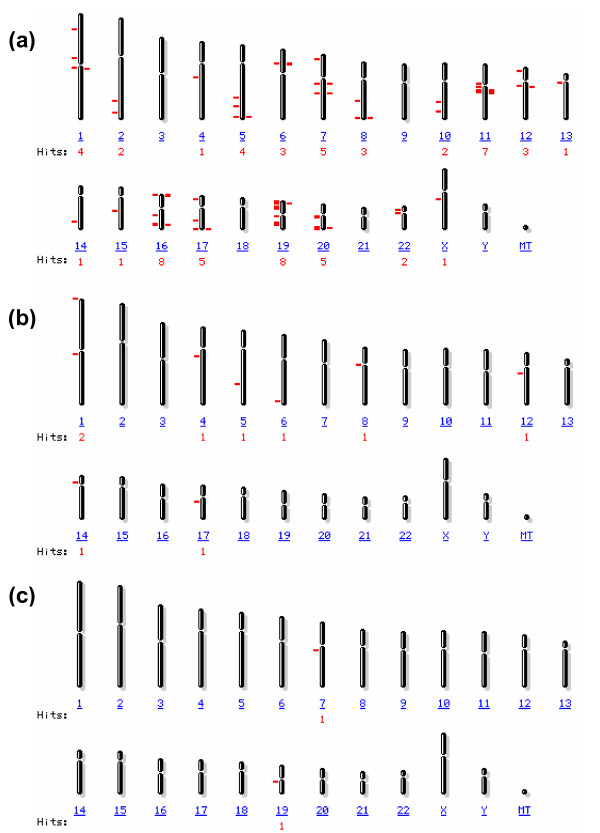
**Chromosomal localization of 77 common genes in MCF-7 and ZR75-1 cells**. **(a) **Localization of 66 up-regulated genes, **(b) **localization of 9 down-regulated genes, and (**c**) localization of two oppositely expressed genes (also see Table 2, additional file 2). The number of hits (in red) represents the number of genes expressed in each chromosome (denoted in blue).

Common genes had good correlation. More genes were up-regulated (66) compared to the down-regulated (9) ones and 2 genes had opposite expression (Figures 1, 2, see additional file 4). For the GO term BP, the highest gene distribution was seen in physiological, cellular and cellular physiological processes (~45–62%), followed by macromolecule metabolism and biosynthetic processes (~10–50%), while only 5–6% genes were used for sugar metabolism and protein folding. For MF, genes regulating catalytic and transporter activities were highest (19–32%) and 5–18% genes were in other categories. About 38–57% common genes constituted of the intracellular, organelle, membrane-bound organelle and cytoplasmic components (CC), 5–18% genes were distributed in other categories, including the mitochondrial electron transport chain (Figure 1d). Similarities being evident in physiological processes (BP), catalytic activity (MF), or the intracellular and organelle components (CC), there were differences in gene distribution in metabolic processes or protein folding (BP), in transporter activities and electron transport functions (MF), and in specific cellular compartmental components (CC). This indicated difference in common gene distribution within the GO terms (Figure 1d). For the up-regulated common genes, most genes (≥ 5) were localized to chromosomes 7, 11, 16, 17, 19, and 20. Chromosomes 11, 16 and 19 harbored genes with highest expression (mostly in ZR75-1). Chromosomes 1, 4, 5, 6, 8, 12, 14, and 17 harbored 9 down-regulated genes and chromosomes 7 and 19 harbored the 2 oppositely expressed genes (Figure 2; see additional file 2, Table 2).

### Gene expression and GO show distinct cluster signatures (CS) in MCF-7 and ZR75-1

**Figure 3:**
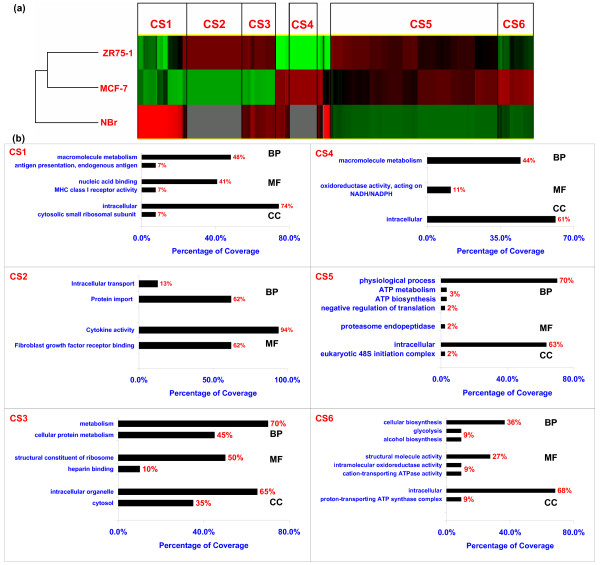
**A cluster of 263 differentially expressed genes depicting distinct expression profiles of MCF-7, ZR75-1, and NBr**. **(a) **These genes formed six distinct cluster signatures (CS) between the three SAGE libraries. Each row in the cluster corresponds to a SAGE library. Percent coverage of genes within the GO terms in each CS is shown lower to the cluster. In the cluster, intense **red **color correlates with high expression, **black **indicates low expression, **green **is negative expression, and **grey **is missing expression (genes and their corresponding Unigene IDs are shown in Figure 1A a, b, additional file 3). **(b) **Graphs highlighting the salient distribution of genes within each of the GO terms, biological process (BP), molecular function (MF), and cellular component (CC) in the six distinct CSs (a detailed distribution of genes within the GO terms in each CS is shown in Figure 1A c: CS1-CS6, additional file 3). DAVID did not classify any genes for CC in CS2.

Based on differential expression patterns, genes forming distinct CSs were annotated for GO terms using Cluster and DAVID tools for 263 significant genes. We identified six distinct CSs (CS1-CS6), which correlated with genes distributed in pathways in both cell lines (Figure 3; also see Figure 1A a,b,c, additional file 3). Among other GO terms, the striking feature of CS1 was a marked down-regulation of genes controlling immune response, protein biosynthesis and macromolecule biosynthesis in MCF-7 and ZR75-1 as reflected in the process (BP) and function (MF) gene utilization. ZR75-1 cells mainly had elevated gene expression in CS2 for protein import (BP) and cytokine activity (MF); in addition, ZR75-1 cells also had elevated expression of genes involved in transport processes and increased growth factor activity in CS2. In CS3, genes related to protein metabolism were differentially elevated, whereby ZR75-1 cells had increased gene distribution in protein biosynthesis (MF) as well as in the cellular components related to protein biosynthesis (CC) relative to MCF-7. On the other hand, in CS4, MCF-7 cells had a relatively up-regulated gene distribution in energy related functions (MF) compared to ZR75-1 cells. Collectively taken, physiological, cellular, transport, metabolism, were increased in both breast cancer cells, which was in agreement with GO distribution in individual and common genes (Table 2, Figure 1d). In CS5, in contrast to NBr, breast cancer cells had an increased gene distribution for physiological and cellular processes, and metabolism (BP). A decreased gene distribution was seen for oxidative phosphorylation, transport, apoptosis and nucleic acid metabolism (ATP/GTP metabolism and synthesis) respectively (BP). For MF, the highest gene distribution was seen in catalytic activity, protein binding, and transporter activity and lesser distribution was seen in cytochrome c oxidase and endopeptidase activity. While there was an increase in gene distribution for intracellular components including mitochondria, decreased distribution was seen in the electron transport chain and cellular protein metabolism components (CC). In contrast to ZR75-1, CS6 had up-regulation of genes regulating sugar metabolism (glycolysis/gluconeogenesis) and ATP metabolism in MCF-7 cells (BP), transporter activity (MF) and related cellular compartments, with the exception of ribosome (CC). Based on GO term analysis, we compared pathways (electron transport, glycolysis, proteosome, ribosome) with fold changes (up/oppositely expressed) of common genes and cell line specific pathways.

To compare breast cancer cells with different ER status, we did a cluster of the 263 gene dataset of MCF-7 and ZR75-1 with a highly aggressive ER(-)ve cell line MDA-MB-435 [19]. Despite the heterogeneity among the consensus genes, we found a down-regulated expression of genes of the electron transport chain (complex I, IV, V) and protein synthesis (genes expressing ribosomal proteins), and up-regulation of some genes of the glycolytic pathway in the MDA cells (Figure 1Bi, ii, see additional file 3). Also, the down-regulation of luminal keratin 18 and NME1 in MDA cells was similar to ZR75-1, but different from MCF-7 (Figure 1Biii, see additional file 3). We found no correlation in the 115 consensus genes between the E2 deprived and cycling MCF-7 cells (Figure 1C, see additional file 3) and were unable to compare with cycling ZR75-1 cells due to the unavailability of SAGE/MA gene expression data.

### Correlation of gene sets in MCF-7/ZR75-1 with breast tumor tissues

**Figure 4:**
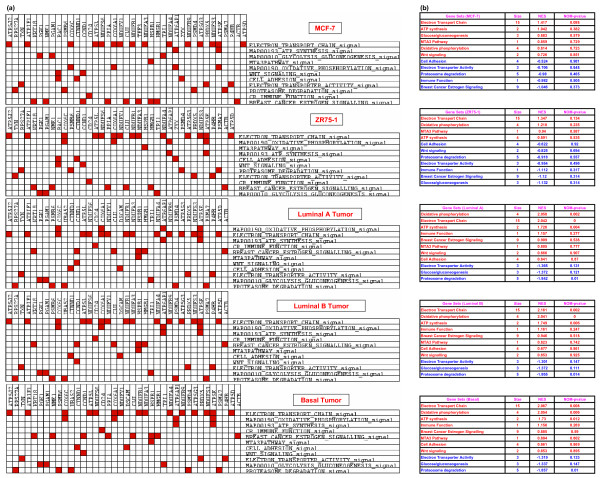
**Enriched Gene Sets for compared pathways operating in MCF-7 and ZR75-1 cells correlated to ER(+)ve and ER(-)ve breast tumor tissues**. **(a) **Unclustered heat map of leading edge subsets in all five groups as analyzed by the GSEA. Rows represent gene sets and columns are genes. This matrix is not clustered, but shows the distribution of genes in each of the gene sets in MCF-7 and ZR75-1 cells in correlation to the ER(+)ve luminal (A and B) and ER(-)ve basal tumors. **(b) **Enriched gene sets as identified by the GSEA tool. The primary statistic denoting gene enrichment is given by the normalized enrichment score (NES), size is the number of genes in the gene set (in a few cases, the actual number of genes analyzed by the GSEA tool was different), NOM p-val is the nominal P value. For individual gene expression, refer to the cluster heat map (Figure 1D, additional file 3).

To correlate the results in MCF-7 and ZR75-1 cells to ER(+)ve (luminal A and B) and ER(-)ve basal breast tumor tissues, we compared eleven gene sets from pathways with distinct differential gene expression by GSEA analysis. Despite the differences in cell growth conditions (E2 deprivation), global gene expression techniques (SAGE, MA), and tissue types (luminal and basal), as shown in the individual heat maps, the compared pathways had almost identical gene enrichment (given by the normalized enrichment score, NES) and distribution, but different gene expression between groups (Figure 4a,b; Figure 1D, see additional file 3). Scores were highest for electron transport chain, oxidative phosphorylation, and ATP synthesis pathways in all groups; ZR75-1 cells resembled breast tumors exhibiting low enrichment scores for glucose/gluconeogenesis pathway. In contrast, MCF-7 cells had higher gene enrichment for sugar metabolism, in agreement with cluster analysis (CS6, Figure 3). The non-enriched pathways mainly included breast cancer estrogen signaling, electron transporter activity, and proteosomal degradation in all groups; cell lines had a down-regulated immune function and cell adhesion in contrast to the tumor cells. Enrichment for Wnt signaling and MTA-3 pathways was relatively identical in all groups. Individual inspection showed that genes of the electron transport chain were down-regulated in all groups, but to a higher degree in tumors compared to cell lines, especially in basal-like tumor. Genes of the breast cancer estrogen signaling pathway were progressively down-regulated from ZR75-1 to luminal B tumors and in basal tumors there was a total absence of expression. This trend of gene expression was also seen for immune function, Wnt signaling, MTA-3, and cell adhesion pathways. A progressively down-regulated gene expression from cell lines to tumors, though heterogeneous, was seen in proteosome degradation and glucose/gluconeogenesis pathways (Figure 4). While NES and gene expression were similar in luminal and basal tumors, there were differences in the genes expressed for pathways such as breast cancer estrogen signaling, immune function, and electron transporter activity. This could be attributed to the heterogeneity of breast tumors and to the limited number of consensus genes chosen by the GSEA tool. Overall, ZR75-1 shared a trend of similarity with tumors.

### Energy production and sugar metabolism

**Figure 5:**
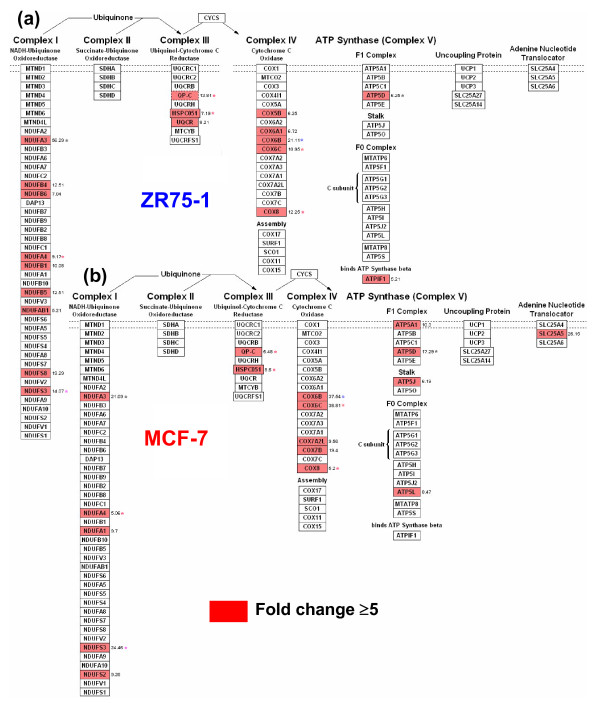
**A GenMAPP view of energy production pathway in MCF-7 and ZR75-1 cells**. Common genes are denoted by an* and significant genes with a fold change of ≥5 and ≤-5 were taken. Genes in the GO list were rearranged with GenMAPP to depict electron transport in **(a) **ZR75-1 and **(b) **MCF-7 cells.

**Figure 6:**
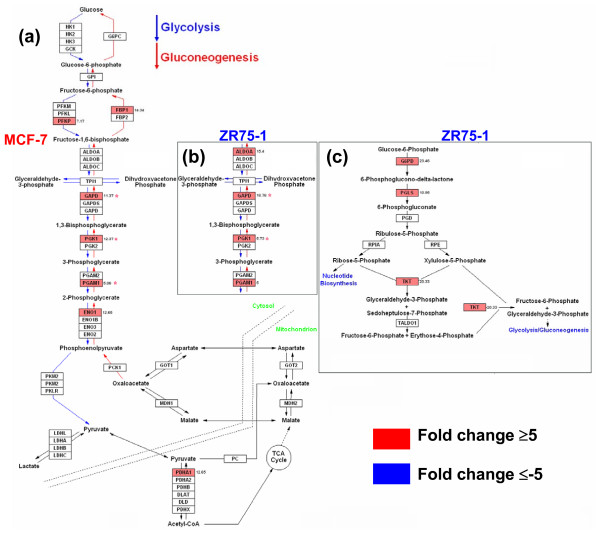
**Sugar metabolism pathways in MCF-7 and ZR75-1 cells profiled by GenMAPP**. Common genes are denoted by an * and significant genes with a fold change of ≥5 and ≤-5 were taken. Genes in the GO list were rearranged with the GenMAPP to depict the glycolysis/gluconeogenesis pathways in **(a) **MCF-7, **(b) **ZR75-1 cells and the **(c) **pentose phosphate (hexose monophosphate shunt, HMS) pathway in ZR75-1 cells.

Given the individual similarity of gene distribution for the electron transport/oxidative phosphorylation pathways, the percent utilization and the number of up-regulated genes (19 versus 17 genes) was more in ZR75-1 than MCF-7 (Figures 1c, 5). In complexes I and III, ZR75-1 up-regulated more genes, and in complex IV, both cells up-regulated 5 genes; among the 3 common genes, MCF-7 cells had a higher expression of ATP5D. Gene utilization doubled in MCF-7 cells in complex V compared to ZR75-1 (4 and 2 genes). MCF-7 cells used ATP5A1, ATP5J (10.3 fold) and SLC25A5/ANT2 (adenine nucleotide translocator 2; 26.2 fold) in complex V. Glucose metabolism was higher in MCF-7 exhibiting a high glycolysis rate (CS6, Figures 3b, 6a) while ZR75-1 showed conservation (4 versus 7 genes). GAPDH, PGK1 and PGAM1 were common (Figure 6b); un-induced ZR75-1 highly expressed ALDOA and GAPDH, whereas E2 induces lower expression [8]. ZR75-1 cells preferably used the HMS pathway for glucose metabolism (Figure 6c). Besides MCF-7, ZR75-1 and all tumors had negative gene enrichment for sugar metabolism (Figure 4).

### Ribosome pathway and the proteosomal degradation pathway

**Figure 7:**
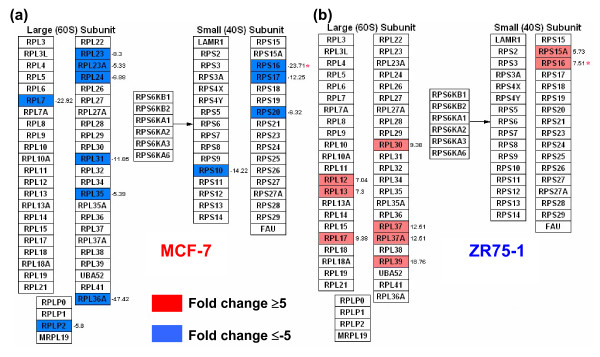
**The ribosome pathway in MCF-7 and ZR75-1 cells**. **(a) **All 12 genes in this pathway were down-regulated (GenMAPP) in MCF-7. **(b) **In contrast, all the 9 genes were up-regulated in ZR75-1 cells. The only common gene (*) in the small subunit (40S) is RPS16, which was down-regulated (-23.7 fold) in MCF-7 and up-regulated (7.5 fold) in ZR75-1 cells. The up-regulated ribosomal genes in ZR75-1 indicate increased protein synthesis in these cells in contrast to MCF-7 cells (also refer to CS3, Figure 3b).

**Figure 8:**
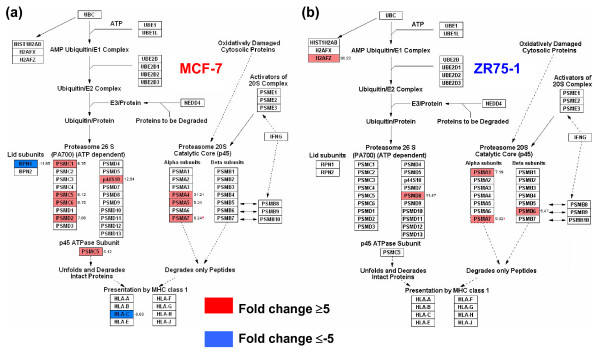
**The proteosomal degradation pathway**. The pathway in **(a) **MCF-7 and **(b) **in ZR75-1 cells. MCF-7 cells had a higher gene usage and selected genes which encode for the ATPase subunit proteins in the 19S regulator (PSMC1, PSMC5, PSMC6), versus no change in ZR75-1 cells. PSMA7 (alpha subunit, 20S catalytic core) was the only common gene with an identical fold change. The histone family member protein encoding gene, H2FZ was up-regulated in ZR75-1 cells.

Though GO terms related to protein biosynthesis and metabolism was decreased in both cells relative to NBr (CS1), ZR75-1 had increased gene utilization relative to MCF-7 cells (CS3, Figure 3). Comparison with other pathways showed that the ribosome pathway was distinct because all 9 genes were up-regulated in ZR75-1, versus all 12 down-regulated genes in MCF-7 (Figure 7). More genes, including the oppositely expressed common gene RPS16, were in the 60S, but not in the 40S subunit. In the proteosome pathway, besides more gene utilization, there was a difference in genes expressed by MCF-7 from ZR75-1 (Figures 1c, 8). MCF-7 cells selectively up-regulated 3 of 6 genes encoding for ATPase subunits (PSMC1, 5, 6) and a single non-ATPase subunit coding gene, PSMD2 in the 19S regulatory subunit versus none in ZR75-1 cells. A single gene (19S regulator, a part of the 26S complex) encoding the non-ATPase subunits were up-regulated in MCF-7 (p44S10/PSMD6) and ZR75-1 (PSMB8). In the alpha subunit (20S catalytic core), MCF-7 up-regulated 3 versus 2 genes by ZR75-1, and PSMA7 was common. In the beta subunit (20S catalytic core), ZR75-1 had a higher expression of PSMB6 (Figure 1Aa, see additional file 3) than MCF-7. MCF-7 also up-regulated PSMC5, which encodes for another ATPase subunit and down-regulated HLA-C (-8.7 fold, presentation by MHC class I) and lid associated RPN (-11.9 fold). ZR75-1 cells up-regulated H2FZ, which encodes for a histone family member protein. Comparison of the proteosome degradation pathway by GSEA analysis showed a low gene enrichment score for both cell lines and breast tumors (Figure 4). Of the 5 consensus genes, PSMD4 and PSMA7 were highly expressed in cell lines, but absent in tumors. High PSMB6 expression was ubiquitous, UBA52 was low to absent, and RPS27A expression was heterogeneous among the groups (Figure 1D, see additional file 3). In both cells, the fold change was <5 for PSMD4 and PSMB6 (compared to NBr).

### Other individual and common pathways in MCF-7 and ZR75-1 cells

Some pathways operating in MCF-7 were MAPK, tight junction, TGF beta signaling, Wnt signaling, Toll like receptor, steroid biosynthesis and others (Figure 1c; see additional file 4, Table 1a and Figures 3, 4, 5). The Wnt signaling pathway showed a relatively high enrichment by GSEA analysis in MCF-7 cells similar to luminal and basal tumors, but not ZR75-1 cells (Figure 4). ZR75-1 cells highly expressed 4 genes (GAPD, ALDOA, HSPB1, MTA1) in the MTA-3 (BIOCARTA) pathway (Figure 1c; Table 1b, see additional file 1; Figure 6, see additional file 4) cells. However, GSEA analysis selected the HSPB1 gene only in the MTA-3 pathway for tumors and cell lines; the gene was down-regulated in luminal and basal tumors (Figure 1D, see additional data file 3). In the tight junction pathway claudin4 was highly down-regulated (-92.5) and Rab13 was highly up-regulated (27.8) (Figure 3, see additional file 4) among the 7 genes used in this pathway. In the retroviral genome replication pathway, IL8 (-43.5) was significantly down-regulated in ZR75-1 while MCF-7 down-regulated TNIP1 (-7.5). Immune function was negatively enriched in both cell lines (GSEA). Both cells had the insulin signaling, focal adhesion and ATP synthesis pathways (Figure 1d; see additional file 1, Table 1).

## Discussion

In this *in silico *study, gene expression and enrichment analyses, pathways, and chromosomal localization of genes reflect integral metabolic differences between the two ER(+)ve luminal A type human breast cancer cell lines, MCF-7 and ZR75-1 cultured under E2 deprivation [4,10]. As this study is centered around two well-characterized cell lines, which share substantial global similarities in their transcriptomes with ER(+)ve breast tumors, we did multiple comparisons to demonstrate similarities and differences with cell lines and tumors with different ER status [20].

Comparison of gene expression of MCF-7 and ZR75-1 cells with basal-like ER(-)ve MDA-MB-435 cells or cycling MCF-7 cells indicate that differences may be a result of ER status, presence/absence of E2, or global gene expression techniques (SAGE, MA). Comparison with cycling MCF-7 cells showed opposite expression of certain genes (not shown), which indicates that gene expression is more likely a result of the presence/absence of E2 in the growth environment rather than the use of different gene expression techniques. When compared to the MDA-MB-435 cells, ZR75-1, but not MCF-7 cells, were found to share similarity in the selectivity of genes of electron transport for energy minimization. For genes related to breast cancer estrogen signaling, GSEA analysis showed a progressive down-regulation of keratin 18 and NME1 from luminal A to basal tumors, which also correlated with ER(+)ve MDA-MB-435 cells; low expression of these genes correlates to poor prognosis and metastasis [21,22]. ZR75-1 cells also have a low expression of these genes, indicating a similarity towards the ER(-)ve phenotype. The MDA cells differed from ZR75-1 cells in having a down-regulated expression of protein synthesis genes and an up-regulation of glycolytic genes; however, down-regulation of glycolysis has been observed in a highly aggressive S100A7 transfected MDA-MB-231 cells [23]. While these differences may be attributed to a different ER status, it is evident that ZR75-1 cells exhibit some characteristics of the ER(-)ve phenotype. Comparison by GSEA showed that all the compared pathways were present in both cell lines, however, ZR75-1 had a greater overall similarity with luminal and basal tumors among the consensus genes.

An interesting facet in these cell lines was the disproportionate contribution of some chromosomes, such as 11, 16, and 19, harboring some of the highly up-regulated common genes. Some of these genes are involved in metal ion binding (PPP1CA), electron carrier and transport activity (CYBA), ATP binding and nucleotide synthesis (NME3), oxidative phosphorylation (NDUFA3), and electron transport (COX6B1) as annotated by DAVID. Interestingly, except for COX6B1, all 3 genes (CYBA, NME3, NDUFA3) were highly up-regulated in ZR75-1 indicative of efficient energy utilization. We found that 67–68% of the common genes were localized to the long arm q in all chromosomes, among which chromosomes 11, 16, and 19 expressed most genes. The amplification and co-amplification with other chromosomes of 11q13 region of chromosome 11 is relatively frequent in breast tumors and overexpression of CCND1 (cyclin D1) and PPP1CA is also seen [24,25]. In the q arm in chromosome 11, CCND1, PPP1CA and 5 other genes contributed to 71% of genes expressed in this chromosome (Table 2, see additional file 2). Chromosome 16q suffers frequent loss of heterozygosity (LOH) in sporadic breast cancer; it expresses a significant list of genes which negatively relate to recurrence-free breast cancer survival [26,27]. Chromosome 19, on the other hand, suffers frequent LOH in its short arm p, which may cause de-regulated expression of more genes in the p arm (this study) [28]. In ZR75-1, some up/down-regulated genes in other chromosomes regulating critical functions such as lipid utilization and binding (FASN, StarD10; chromosomes 17 and 11), ion transport (ATOX1, chromosome 5), and cell survival (HSPB1, NF-κB; chromosomes 7, 14) may also impart aggressiveness to these cells [5,29-32]. Interestingly, FASN, StarD10, HSPB1, NF-κB were down-regulated in MDA-MB-435 cells, constituting a major difference with these ER(+)ve cells. HSPB1, a component of multiple pathways, was also down-regulated in all breast tumors.

Individual GO and CS GO (CS6) showed prominence for glucose metabolism in MCF-7 cells. ZR75-1 cells used the HMS pathway to generate NADPH, which maintains glutathione in a reduced state. Reduced glutathione protects the intracellular sulfhydryl groups to preserve cellular integrity from oxidative stress in cancer and may be protective to the ZR75-1 cells [33]. MCF-7 cells, unlike ZR75-1 and breast tumors, had a high glycolytic rate, which correlated with a high expression of SLC25A5/ANT2 (Complex V, electron transport chain). SLC25A5 imports glycolytic ATP into mitochondria and the overexpressed gene promotes aggressiveness, cell survival, and arrests cell proliferation when inhibited [34]. Increased expression of ANT2 may be a feature of ER(+)ve breast tumors with a high glycolytic rate (as MCF-7), and absence of ANT2 expression in ZR75-1 supports this notion. In the cell lines, gene utilization in the electron transport chain was similar in the common genes (Complex I-IV) although the usage of COXC subunits (Complex IV) and a higher F1 gene utilization (Complex V) was different in MCF-7 cells. FI genes encode for mitochondrial ATP synthase subunits, which was consistent with the moderately increased expression of ATP5D in ZR75-1 cells. In cell lines and tumors, the electron transport chain/oxidative phosphorylation pathway had the highest individual gene utilization and enrichment, although there was decreased gene similarity in the energy associated GO terms. Given the dissimilarity of the growth conditions of study for cell lines (E2 deprivation) with tumors, we found differences in the individual gene expression of complexes I, IV, and V respectively. Negative gene enrichment and a progressive down-regulation (total absence of expression in basal tumors) of genes of the electron transporter pathway from MCF-7 to tumors (lower in ZR75-1 than MCF-7, CS4) indicate a common energy conservation strategy.

In contrast to energy conservation, ZR75-1 cells demonstrated elaborate protein synthesis mechanisms (ribosome pathways, CS3). These cells have an increased ribosomal gene expression, constituting a major difference with the MCF-7 cells. Notably, the only common up-regulated gene, RPS16 in ZR75-1 (down-regulated in MCF-7) correlates with its overexpression in breast, colon, prostate, liver, and pancreatic tumors [35,36]. The increased ribosomal gene expression (and protein synthesis) as seen in ZR75-1 cells, is a feature of invasive and metastatic breast cancers but not *in situ *cancers [6]. In the proteosome pathway, MCF-7 selectively up-regulated more genes encoding for ATPase subunits (PSMC1, 5, 6) than the non-ATPase subunit coding genes (PSMD2) in contrast to none by the ZR75-1 cells. The up-regulation of only p44S10/PSMD6 (19S non-ATPase subunit) by MCF-7 is of significance as the encoded protein subunit possesses ATPase activity and ATP-dependent proteolytic function and has been shown to have increased copy number in cutaneous cancers and MCF-7 cells [37]. In contrast, ZR75-1 up-regulated PSMD8, whose protein has a non ATP-dependent proteolytic activity [38]. PSMA7, the gene expressed in common in MCF-7 and ZR75-1, encodes for the core alpha subunit regulating the transcription factor hypoxia-inducible factor-1α, which produces hypoxic responses in breast tumors [39]; however, the absence of PSMA7 expression in luminal and basal tumors may be indicative of either a heterogeneity of expression in breast tumors or its specific expression in E2 deprivation. This is also true of PSMD4 whose expression was absent in tumors, but high in cell lines. Interestingly, the expression of the ubiquitin (Ub) gene UBA52, whose protein is a part of the proteoasome/Ub complex, was low to undetectable in all groups. This indicates down-regulated ATP-dependent degradation of Ub-conjugated proteins in breast tumors; this was in contrast with an increased ATP/Ub-dependent protein degradation in renal cancer [40]. However, cell lines and tumors had an up-regulated expression of PSMB6, whose protein is also involved in ubiquitin-dependent protein catabolism. The reason for choice of PSMB6 is unclear in breast cells/tumor, but high expression is also found in thyroid cancer although not much is known about its role in carcinogenesis [41]. The highly up-regulated gene PSMA4 in MCF-7, encoding for an immunoproteosome, is involved in the processing of class I MHC peptides (HLA-C was down-regulated in MCF-7) and possesses threonine endopeptidase activity. RPN1 (lid subunit) mediates ubiquitin-like domain binding to this proteasome for protein amino acid glycosylation [42]. Down-regulated RPN1 agrees with decreased protein synthesis (and hence post-translational modification) in MCF-7 (ribosome pathway). Interestingly, ZR75-1 cells highly up-regulated H2AFZ (proteosome pathway), a member of the histone H2A family and a replication-independent gene [43]. Lack of H2AFZ is lethal in mouse embryos, hence this gene may be protective in E2 deprivation for the ZR75-1 cells [44].

MCF-7 cells also mobilized other pathways such as MAPK, tight junction, TGFβ signaling, Wnt, and shared common pathways as insulin and focal adhesion signaling pathways with ZR75-1. The MAPK pathway is active in breast and other cancers, and it also activates other pathways such as the insulin receptors/insulin-like growth factor receptors (IGFR) pathway [45]. The insulin signaling pathway influences breast tumor growth, proliferation, transformation and survival [46]. Moreover, IGF can activate the ER through the Ras-MAPK pathway and enhance its transcriptional potential without E2 binding [47]. α-Catenin/CTNNB1 belongs to the E-cadherin-catenin family of adhesion molecules, and it is a key regulator of the Wnt signaling pathway (and TGFβ signaling pathway). CTNNB1 links the β/γ-catenin-cadherin complex to the cytoskeleton to make a functional E-cadherin complex. Abnormal E-cadherin and CTNNB1 positively correlates with high grade ductal carcinomas, lymph node metastasis and poor survival [48]. The down-regulated expression of the transcription factors STAT3 and JUN in the TGFβ signaling/Wnt pathway indicates a repression of gene expression of the un-induced MCF-7 cells. P120 catenin or CTNND1 also belongs to the E-cadherin complex. Immunodetectable diffuse cytoplasmic localization of CTNND1 is found in lobular carcinoma, whereas ductal breast carcinoma retains the dominant membrane immunostaining pattern [49]. In our GSEA analysis, we found that CTNND1 was ubiquitously expressed in all breast tumors and MCF-7 and ZR75-1 cells, which made it hard to discriminate the relation of this gene in the cell lines in the light of breast tumors we examined. Immunostaining experiments may discriminate this relationship with precision. Other low expressing genes included ZYX and RAC1 (cell lines) and DSCAM expression was absent in tumors. The expression of claudin4, a tight junction protein, is highly down-regulated in MCF-7 (-92.5 fold), which correlates with weak or absence of expression in grade 1 invasive carcinomas compared to benign breast epithelia and normal breast [8,50]. Claudin4 protein potently inhibits invasion and metastasis in pancreatic cancer cells and therefore a weak/absence of expression in these invasive cell lines is likely [51]. As seen in invasive and metastatic tumors, MCF-7 cells highly expressed Rab13 [6].

Compared to MCF-7 and tumors, GSEA gave a higher enrichment score for ZR75-1 cells for the ER regulated MTA-3 pathway, although GSEA predicted the expression of only one consensus gene, HSPB1 (Hsp27). In this pathway, as annotated by DAVID, ZR75-1 also had an up-regulated expression of MTA1, ALDOA, and GAPDH. Besides the MTA-3 pathway (ZR75-1), HSPB1, an ATP-independent chaperone with the hsp20-like chaperonedomain, operates in multiple pathways like the breast cancer estrogen signaling pathway (MCF-7, ZR75-1). Functionally, HSPB1 inhibits heat shock-induced cellular protein synthesis, increases cell survival, and is a crucial component of oncogenic pathways and it may thus be preferred by ZR75-1 [31]. The MTA proteins, MTA1-3 are components of chromatin remodeling pathways [52]. These proteins form a repressive chromatin complex shutting down the expression of target genes; expression of MTA proteins also increases the risk of cancer susceptibility and may render aggressiveness to the ZR75-1 cells [53]. In the retroviral genome replication pathway (not shown), MCF-7 suppressed TNIP1 (-7.5), but ZR75-1 cells exhibit a greater immune suppression by significantly down-regulating IL8 (-43.5). This agreed with a lesser enrichment score for immune function in ZR75-1 cells compared to MCF-7, a characteristic seen in the aggressive ER(-)ve breast cancer cells and in basal tumors (this study) [23]. On the other hand, MCF-7 cells may gain survival advantage by greater mobilization of the focal adhesion pathway [54].

Relating gene expression to clinical outcome is crucial in understanding the diversity of breast tumors. Clinically, patients with ER(+)ve tumors have a favorable prognosis, even though E2 is a powerful mitogen in receptor-positive cells. However, even patients within the ER(+)ve subtype have a different outcome; patients with luminal A subtype ER(+)ve tumors have a better prognosis and relapse-free survival compared to those with luminal B or C type [55]. This *in silico *study is of significance and is translatable to human biology as the evidence presented here shows that differences exist between breast cancer cells with similar ER status (luminal A type) and also for the recent finding that MCF-7 or ZR75-1 cell lines are good models to identify important molecular events of ER(+)ve breast tumors [20]. Multidirectional computational analyses in cell line models may thus reflect tumor characteristics which are of importance in predicting patient outcomes even within a narrow subset of tumors (example luminal A subtype).

## Conclusion

This study shows that ZR75-1 cells selectively use genes for energy, protein synthesis and sugar metabolism and other pathways differently from the MCF-7 cells. ZR75-1 cells share characteristics of ER(-)ve breast tumors and cells in certain respects, such as energy minimization and sugar requirements. The specific use of pathways (HMS, ribosome, MTA-3), their down-regulation (immune function), and gene utilization (FASN, STARD10, HSPB1, H2FZ, keratin 18, NME1), probably impart more aggressiveness to the ZR75-1 than MCF-7. Salient differences in these ER(+)ve breast cancer cells require further testing, nevertheless, they may be decisive determinants of treatment outcome and prognosis of ER(+)ve breast tumors.

## Abbreviations

SAGE = serial analysis of gene expression, Microarray = Affymetrix GeneChip MA; GenMAPP = Gene MA Pathway Profiler, DAVID = Database for Annotation, visualization, and integrated Discovery, GSEA = gene set enrichment analysis.

## Competing interests

The author(s) declare that they have no competing interests.

## Authors' contributions

JRD and SM conceived the study. SM conceived the study design, performed biocomputational analysis, data interpretation, and wrote the manuscript. JRD critically reviewed the manuscript, supervised the study and provided funding. Both authors read and approved the final manuscript.

## Pre-publication history

The pre-publication history for this paper can be accessed here:



## Supplementary Material

Additional file 1 The data represents the distribution of individual genes and genes common to both breast cancer cell lines, MCF-7 and ZR75-1 in the major pathways. Click here for file

Additional file 2 The localization of common genes in the chromosomes for the breast cancer cell lines, MCF-7 and ZR75-1. Click here for file

Additional file 4 A representation of the relationship between common genes in both breast cancer cell lines, MCF-7 and ZR75-1. Depiction of some pathways operating in individual cell lines (Figures 1, 2, 3, 4, 5, 6) Click here for file

Additional file 3 Illustration of the expression profiles showing the relatedness of gene signatures between mutual cell lines, MCF-7 and ZR75-1, between MCF-7, ZR75-1, and MDA-MB-435, and between MCF-7, ZR75-1, and breast tumors (Figures 1A–1D) Click here for file
