# Supplementary material for: An integrated analysis of genes and pathways exhibiting metabolic differences between estrogen receptor positive breast cancer cells
Source: BMC Cancer. 2007 Sep 20;7:181. doi: 10.1186/1471-2407-7-181 (PMC2148057; doi:10.1186/1471-2407-7-181)
Supplement: Additional file 4 — A representation of the relationship between common genes in both breast cancer cell lines, MCF-7 and ZR75-1. Depiction of some pathways operating in individual cell lines (Figures 1, 2, 3, 4, 5, 6) [file 1471-2407-7-181-S4.pdf]

Additional File 4: A representation of the relationship between common genes in both the breast cancer cell lines, MCF-7 and ZR75-1. Depiction of some pathways operating in individual cell lines.

Figure 1. A regression plot of fold change of 77 common genes in MCF7 and ZR75-1 cells. Here, we have used regression, a statistical analysis which depicts the relationship between two variables, which in this case were the fold change of common (77) genes between NBr/MCF7 or MCF7/NBr and NBr/ZR75-1 or ZR75/NBr. A plot of fold change was computed using the BioMedCAChe (Bio Medical Computer Aided Chemistry, V 6.2, 2003, Oxford Molecular Limited Fujitsu Limited). The equation as depicted on the plot, gave a value of 0.55 for the regression coefficient r^2.

Figure 2. A plot of fold change of 77 common genes in MCF7 and ZR75-1 cells. The X axis represents fold change of genes and the Y axis consists of gene number.

Figure 3. **(a)** The tight junction pathway operating in MCF7 cells. **(b)** The table at the bottom depicts the fold change of the respective genes. Claudin4 was highly down-regulated and Rab13, a member of the Ras family of oncogenes was highly down-regulated. The heavy polypeptide 9 is not shown in the GenMAPP pathway.

Figure 4. The TGF beta signaling pathway operating in MCF7 cells. The transcription factors STAT3 and Jun are down-regulated, while STAT1was up-regulated. The gene encoding for the cadherin-associated protein, beta-catenin, which was up-regulated (7.6 fold) is common between the tight junction the TGF beta and the Wnt signaling pathways respectively.

Figure 5. The Wnt signaling pathway in MCF7 cells. Besides catenin B1, the transcription factor Jun is common between the Wnt and TGF beta signaling pathways. GenMAPP and DAVID have predicted this pathway for MCF7 cells.

Figure 6. The Down-regulated of MTA3 in ER(-)ve breast tumors pathway was selected by BIOCARTA pathway (a component of DAVID) for ZR75-1 cells. The up-regulated genes are denoted in red.

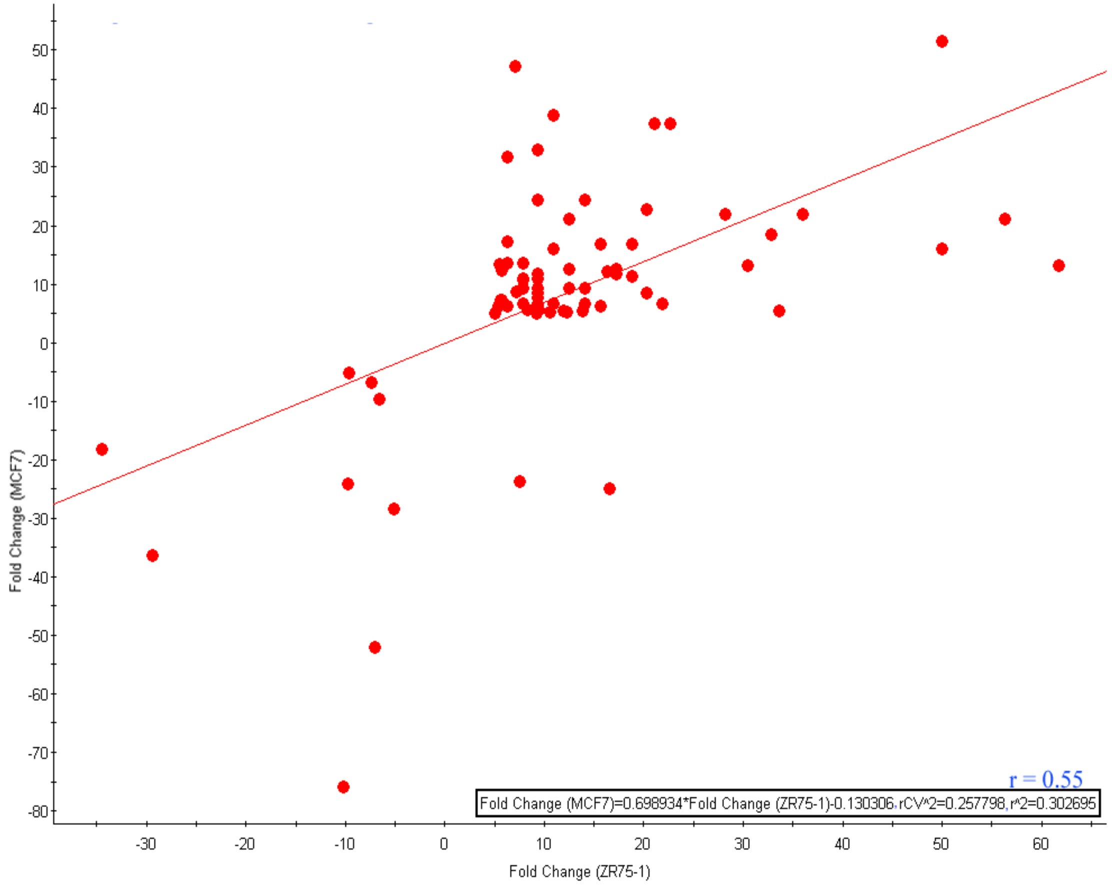

50
40
30
20
10
0
-10
-20
-30
-40
-50
-60
-70
-80
Fold Change (MCF7)
-30
-20
-10
0
10
20
30
40
50
60
Fold Change (ZR75-1)
r = 0.55
Fold Change (MCF7)=0.698934*Fold Change (ZR75-1)-0.130306.rCV^2=0.257798,r^2=0.302695

Figure 1

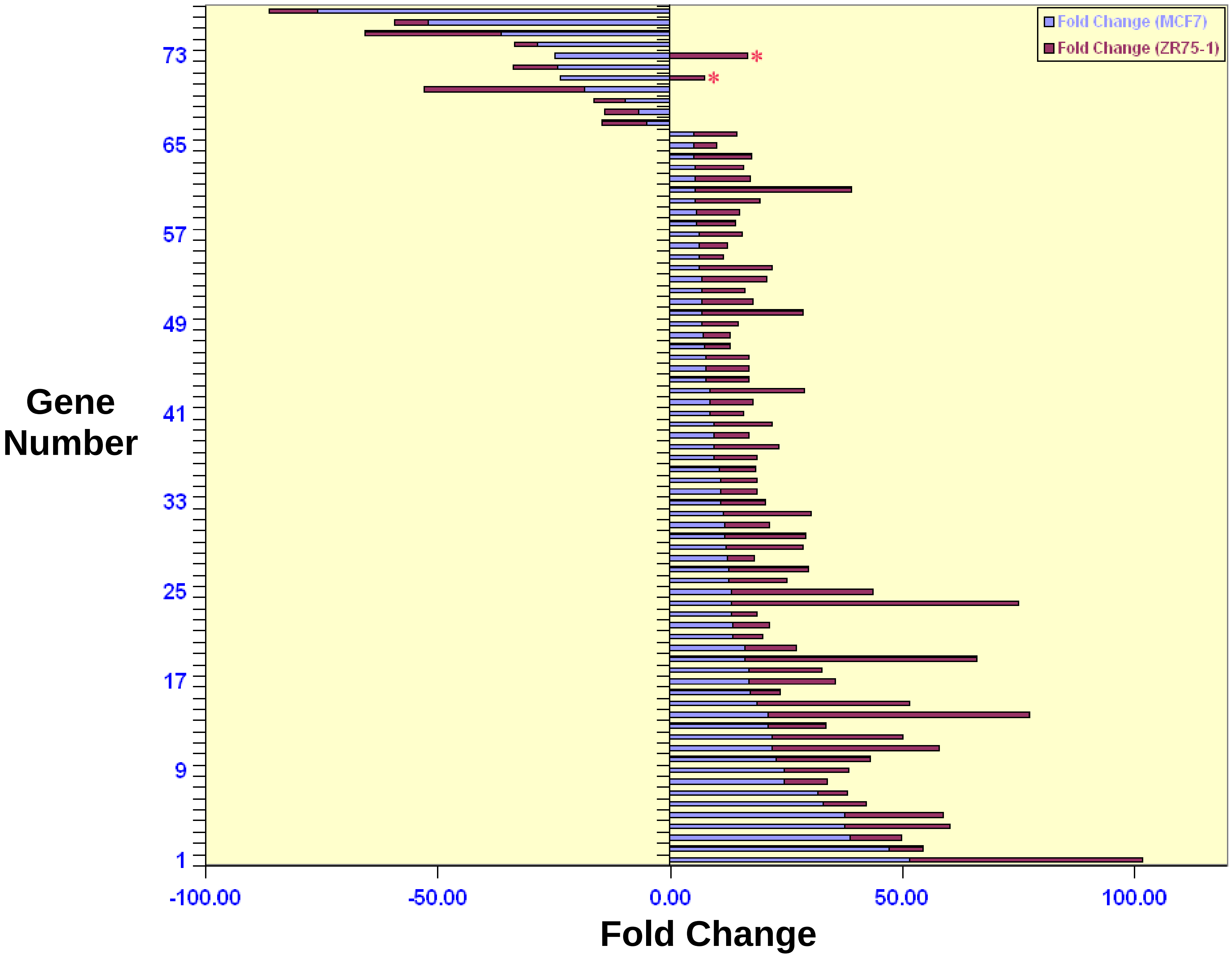

Fold Change (MCF7)
Fold Change (ZR75-1)
Gene Number
73
65
57
49
41
33
25
17
9
1
-100.00
-50.00
0.00
50.00
100.00
Fold Change

Figure 2

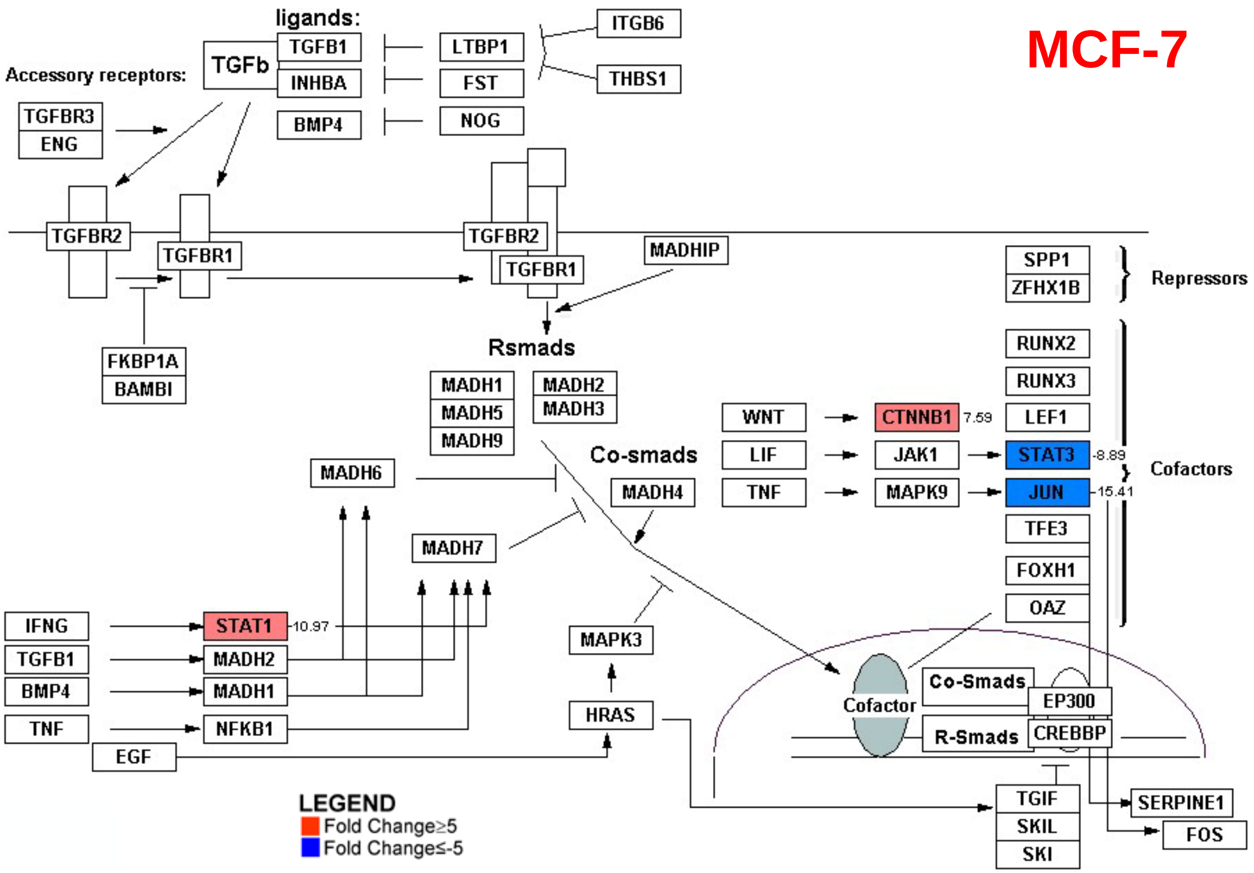
MCF-7
ligands:
TGFb
TGFB1
INHBA
BMP4
LTBP1
FST
NOG
ITGB6
THBS1
Accessory receptors:
TGFBR3
ENG
TGFBR2
TGFBR1
FKBP1A
BAMBI
MADHIP
Rsmads
MADH1
MADH5
MADH9
MADH2
MADH3
Co-smads
MADH4
MADH6
MADH7
SPP1
ZFHX1B
Repressors
RUNX2
RUNX3
LEF1
WNT
CTNNB1
7.59
LIF
JAK1
STAT3
-8.89
TNF
MAPK9
JUN
-15.41
Cofactors
TFE3
FOXH1
OAZ
IFNG
STAT1
10.97
TGFB1
MADH2
BMP4
MADH1
TNF
NFKB1
EGF
MAPK3
HRAS
Cofactor
Co-Smads
R-Smads
EP300
CREBBP
TGIF
SKIL
SKI
SERPINE1
FOS
LEGEND
Fold Change≥5
Fold Change≤-5

Figure 3

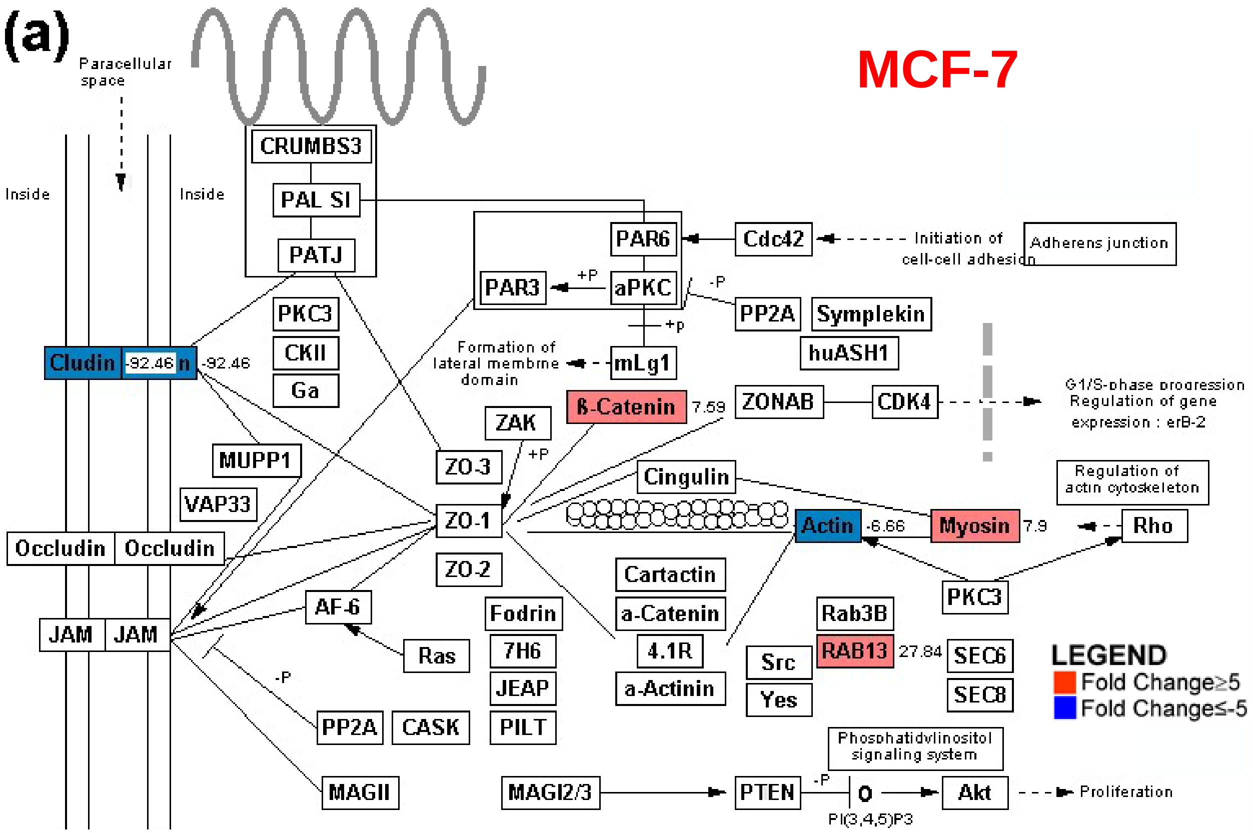

(b)

Gene Name	UniGene ID	Gene Symbol	Fold Change (TPM)
**Genes of the Tight Junction Pathway**			
claudin 4	HS.520942	CLDN4	-92.46
actin, beta	HS.520640	ACTB	-6.66
actin, gamma 1	HS.514581	ACTG1	-6.89
myosin, light polypeptide 6, alkali, smooth muscle and non-muscle	HS.505705	MYL6	7.90
myosin, heavy polypeptide 9, non-muscle	HS.474751	MYH9	-13.04
catenin (cadherin-associated protein), beta 1, 88kDa	HS.476018	CTNNB1	7.59
RAB13, member RAS oncogene family	HS.151536	RAB13	27.84

Figure 4

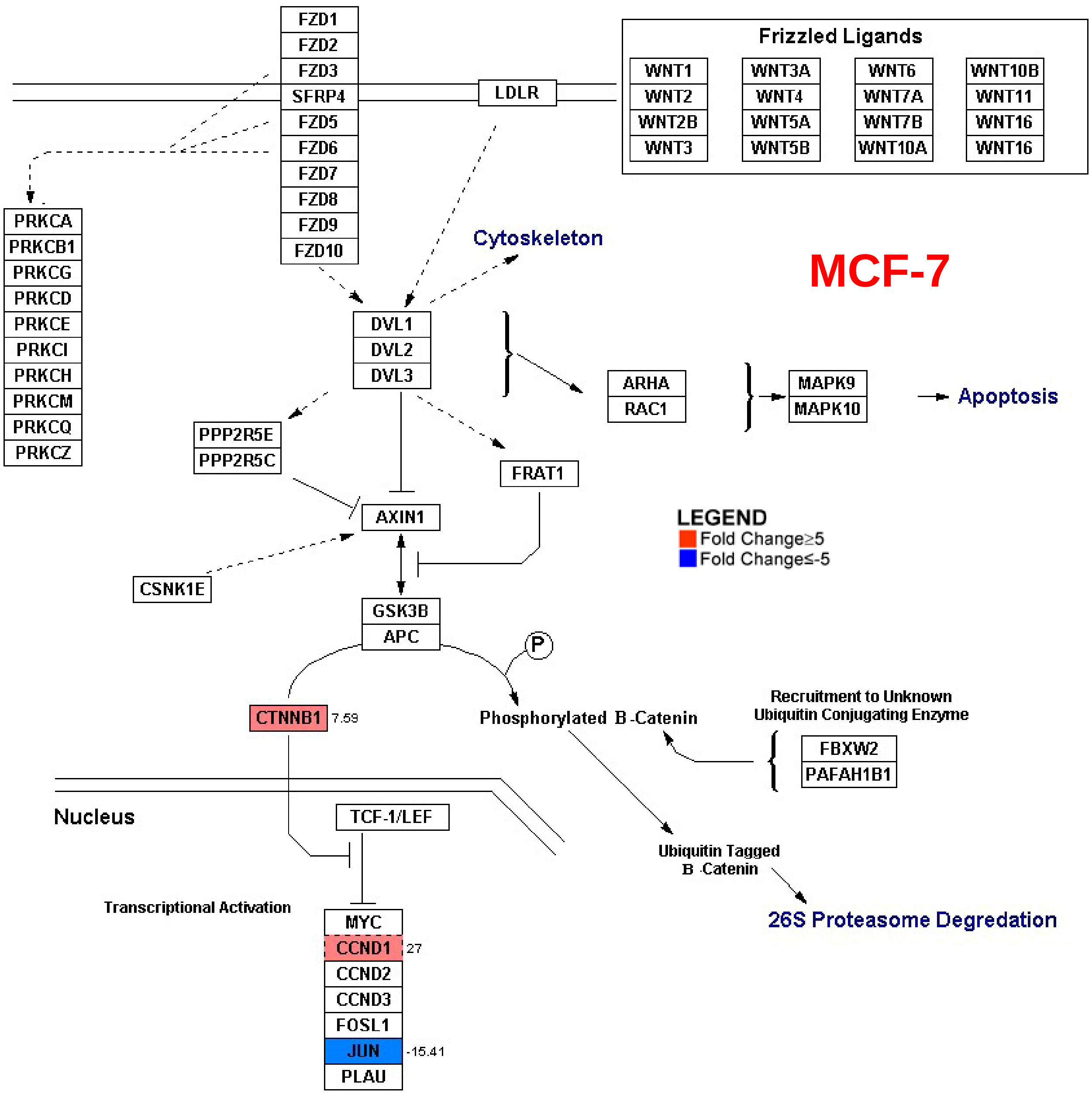
FZD1
FZD2
FZD3
SFRP4
FZD5
FZD6
FZD7
FZD8
FZD9
FZD10
LDLR
Frizzled Ligands
WNT1
WNT2
WNT2B
WNT3
WNT3A
WNT4
WNT5A
WNT5B
WNT6
WNT7A
WNT7B
WNT10A
WNT10B
WNT11
WNT16
WNT16
PRKCA
PRKCB1
PRKCG
PRKCD
PRKCE
PRKCI
PRKCH
PRKCM
PRKCQ
PRKCZ
Cytoskeleton
MCF-7
DVL1
DVL2
DVL3
ARHA
RAC1
MAPK9
MAPK10
Apoptosis
PPP2R5E
PPP2R5C
FRAT1
AXIN1
LEGEND
Fold Change≥5
Fold Change≤-5
CSNK1E
GSK3B
APC
P
CTNNB1 7.59
Phosphorylated B-Catenin
Recruitment to Unknown
Ubiquitin Conjugating Enzyme
FBXW2
PAFAH1B1
Nucleus
TCF-1/LEF
Ubiquitin Tagged
B Catenin
Transcriptional Activation
26S Proteasome Degredation
MYC
CCND1 27
CCND2
CCND3
FOSL1
JUN -15.41
PLAU

Figure 5

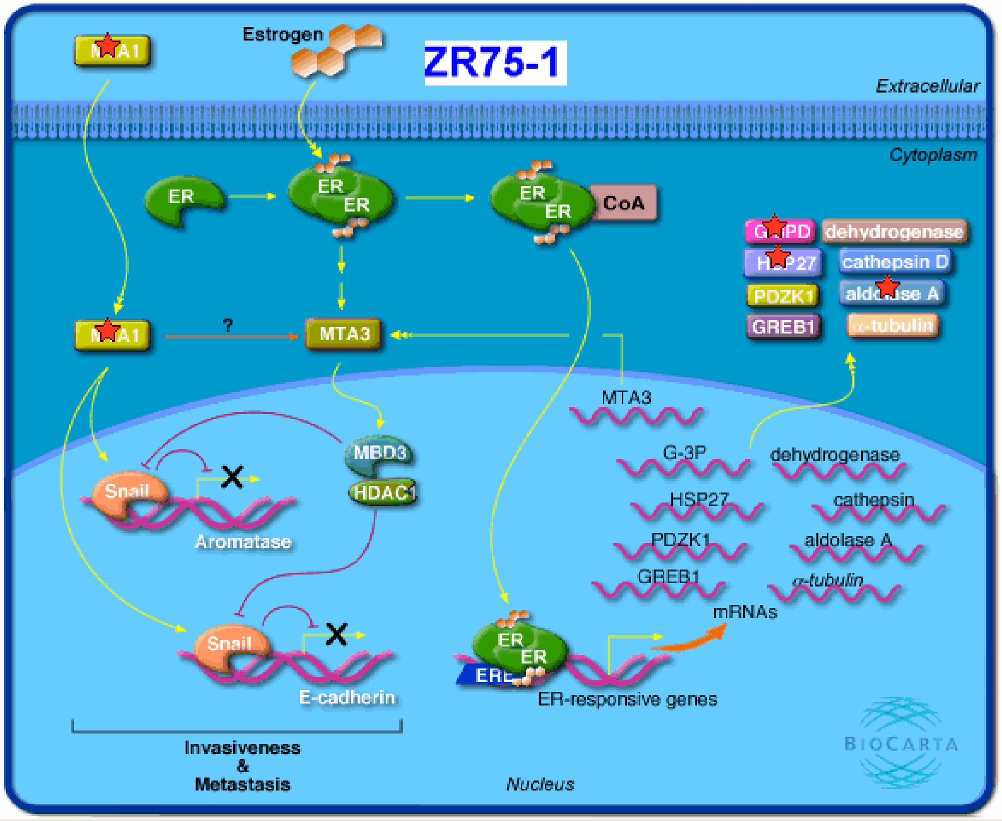

Symbol	DAVID Gene Name	DAVID
HDAC	histone deacetylase 9	1024198
GAPD	*glyceraldehyde-3-phosphate dehydrogenase*	*1500446*
TUBA	dynamin binding protein	757877
ALDOA	*aldolase a, fructose-bisphosphate*	*1168020*
CTSD	cathepsin d (lysosomal aspartyl protease)	1504785
HSP27	*heat shock 27kda protein 1*	*1468826*
PDZK1	pdz domain containing 1	1346811
GREB1	greb1 protein	593324
CDH1	cadherin 1, type 1, e-cadherin (epithelial)	1598136
MBD3	methyl-cpg binding domain protein 3	1629053
SNAIL	snail homolog 1 (drosophila)	1383972
ESR1	estrogen receptor 1	1369260
MTA3	metastasis associated 1 family, member 3	7594
MTA1	*metastasis associated 1*	*810410*

Figure 6
