# Supplementary material for: An integrated analysis of genes and pathways exhibiting metabolic differences between estrogen receptor positive breast cancer cells
Source: BMC Cancer. 2007 Sep 20;7:181. doi: 10.1186/1471-2407-7-181 (PMC2148057; doi:10.1186/1471-2407-7-181)
Supplement: Additional file 3 — Illustration of the expression profiles showing the relatedness of gene signatures between mutual cell lines, MCF-7 and ZR75-1, between MCF-7, ZR75-1, and MDA-MB-435, and between MCF-7, ZR75-1, and breast tumors (Figures 1A–1D) [file 1471-2407-7-181-S3.pdf]

Additional File 3. Expression profiles showing the relatedness of gene signatures between mutual cell lines, MCF-7 and ZR75-1, between MCF-7, ZR75-1, and MDA-MB-435, and between MCF-7, ZR75-1, and breast tumors.

Figure 1A. Hierarchical clustering of significant genes of MCF-7, ZR75-1, and NBr. **(a)** A detailed dendogram showing all the differentially expressed 263 genes (and their corresponding Unigene IDs) between the three libraries, MCF-7, ZR75-1, and NBr respectively. **(b)** Depiction of distinct cluster signatures (CS) between the differentially expressed genes. Each row in the cluster corresponds to a SAGE library. Intense **red** color correlates with high expression, **black** indicates low expression, **green** is negative expression, and **grey** is missing expression (also shown in Figure 3). **(c)** Bar graphs showing the detailed distribution of genes (in the cluster signatures) within the GO terms, biological process (BP), molecular function (MF), and cellular component (CC) in the six distinct CSs. DAVID did not classify any genes for CC in CS2. CS5 (1, 2, 3) and CS6 (1, 2) are divided into separate portions of the GO terms BP, MF, and CC.

Figure 1B. Hierarchical clustering of 215 consensus genes between the ER(+)ve cell lines MCF-7, ZR75-1, and the ER(-)ve MDA-MB-435 cells (refer to Figure 3A) from the respective SAGE libraries (refer to Table 1a). (i) The detailed zoom image of the cluster showing 215 consensus genes in the cluster. (ii) The cluster with the dendogram showing the relationship between the genes from the three cells. (iii) Table highlighting some gene groups (expressed as total TPM) or individual genes (expressed as TPM) of importance to compare between the three cells.

Figure 1C. Regression analysis of tag counts versus SLR (signal log ratio) between SAGE and MA gene expression datasets in MCF-7 cells. Regression is a statistical analysis which depicts the relationship between two variables, which in this case were tag counts and the SLR. The 263 gene dataset of MCF-7 (E2 deprived-SAGE) was compared to the microarray derived gene dataset of MCF-7-NCI, which yielded 115 common genes within the datasets. The contributing differences were attributed to two different global gene expression techniques, namely SAGE and microarray, and two different culture conditions. We found no overall correlation between the two MCF-7 populations, resulting in an r^2 value of 0. In this scatter plot, each circle in the plot corresponds to a transcript. This plot was computed using the BioMedCAChe (Bio Medical Computer Aided Chemistry, V 6.2, 2003, Oxford Molecular Limited Fujitsu Limited). Tag count is related to the SLR value by the following equation:

Log (Tag Count, base2)=0*Log(base 2),SLR+4.42169 rCV^2=-0.000197896 r^2=0

Figure 1D. The heat map showing the clustered genes in the compared eleven leading edge subsets in MCF-7, ZR75-1, luminal A, luminal B, and basal tumors. The expression values are represented as colors, where the range of colors (red, pink, light blue, dark blue) shows the range of expression values (high, moderate, low, lowest). The genes within the pathways selected showed variations in their expression in each of the five groups (also refer to Figure 4a and 4b for the detailed GSEA analysis scores). The tumor tissues used for the GSEA analysis were obtained from the Standford MA database (http://smd.stanford.edu/cgi-bin/data/view). The tumor tissues used here were classified by Sorlie et al., 2001 (Gene expression patterns of breast carcinomas distinguish tumor subclasses with clinical implications, referenced in the main text). The breast tumor tissue data used were, BC_FUMI24-BE (luminal A), BC_FUMI29-BE (luminal B), BC_FUMI41_BE (basal) submitted to the above database (Perou-experimenter).

(a)

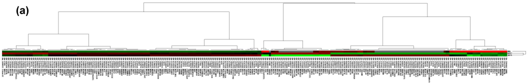

Figure 1A

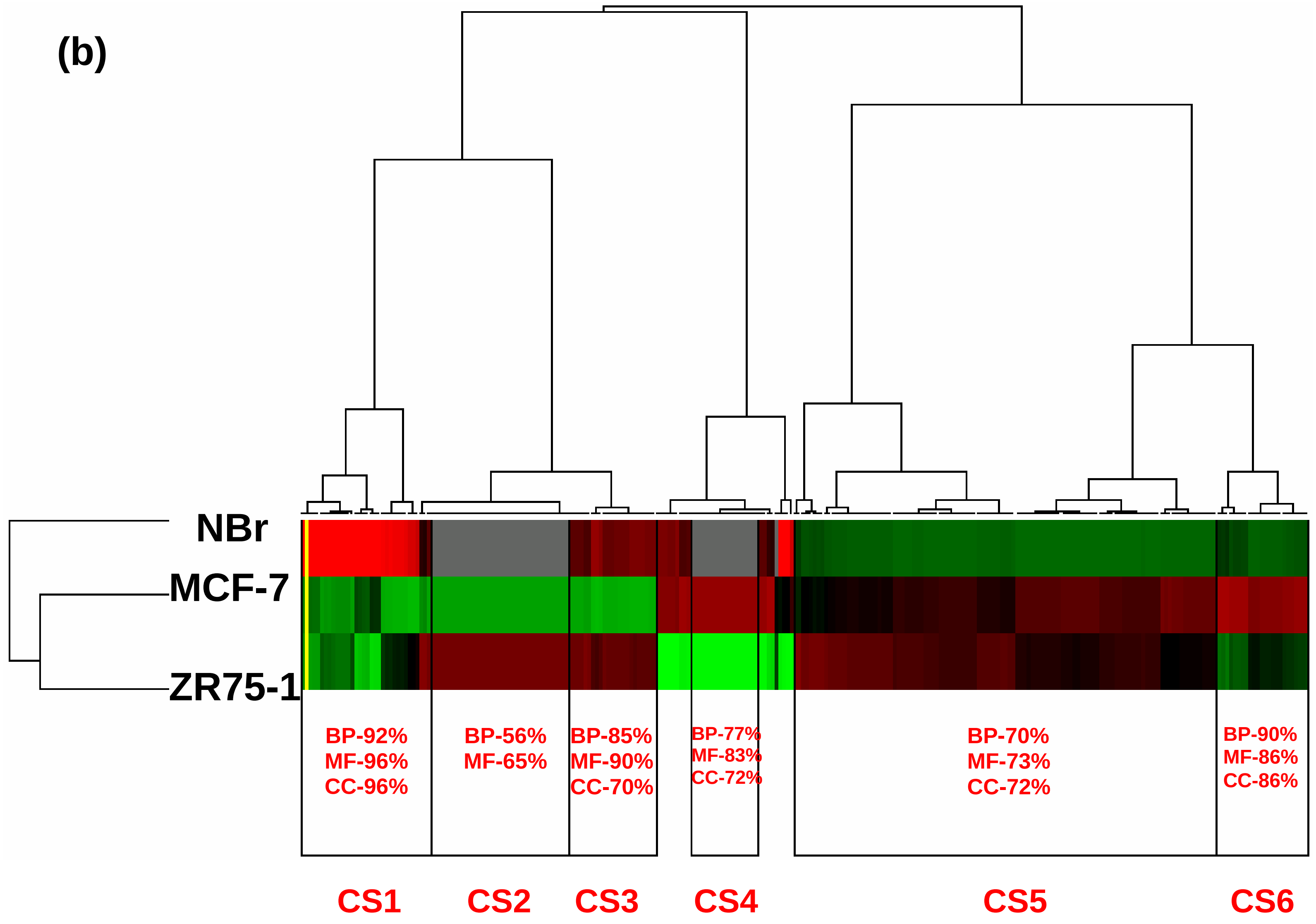
(b)
NBr
MCF-7
ZR75-1
BP-92%
MF-96%
CC-96%
BP-56%
MF-65%
BP-85%
MF-90%
CC-70%
BP-77%
MF-83%
CC-72%
BP-70%
MF-73%
CC-72%
BP-90%
MF-86%
CC-86%
CS1
CS2
CS3
CS4
CS5
CS6

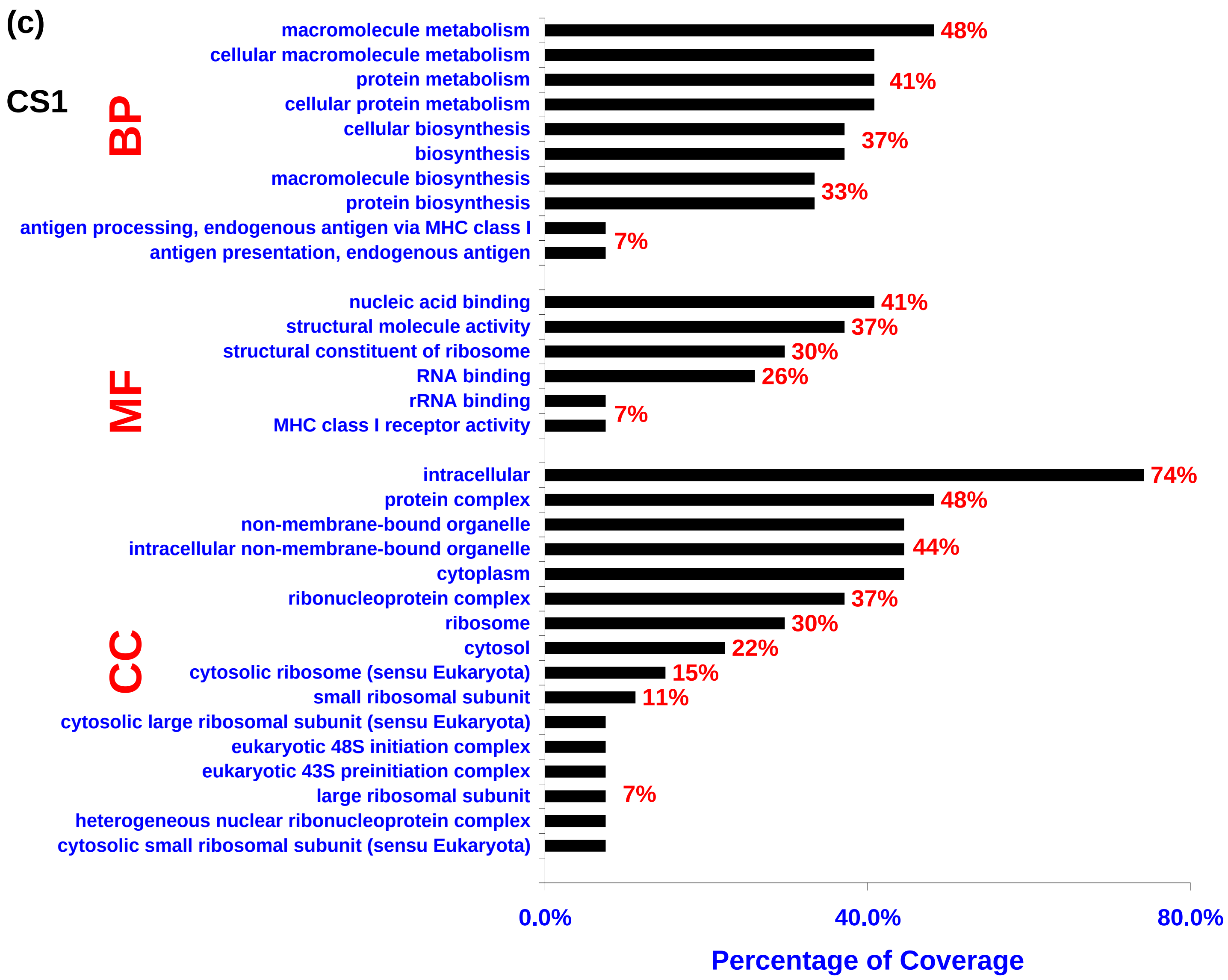
(c)
CS1
BP
MF
CC
macromolecule metabolism
cellular macromolecule metabolism
protein metabolism
cellular protein metabolism
cellular biosynthesis
biosynthesis
macromolecule biosynthesis
protein biosynthesis
antigen processing, endogenous antigen via MHC class I
antigen presentation, endogenous antigen
nucleic acid binding
structural molecule activity
structural constituent of ribosome
RNA binding
rRNA binding
MHC class I receptor activity
intracellular
protein complex
non-membrane-bound organelle
intracellular non-membrane-bound organelle
cytoplasm
ribonucleoprotein complex
ribosome
cytosol
cytosolic ribosome (sensu Eukaryota)
small ribosomal subunit
cytosolic large ribosomal subunit (sensu Eukaryota)
eukaryotic 48S initiation complex
eukaryotic 43S preinitiation complex
large ribosomal subunit
heterogeneous nuclear ribonucleoprotein complex
cytosolic small ribosomal subunit (sensu Eukaryota)
48%
41%
37%
33%
7%
41%
37%
30%
26%
7%
74%
48%
44%
37%
30%
22%
15%
11%
7%
0.0%
40.0%
80.0%
Percentage of Coverage

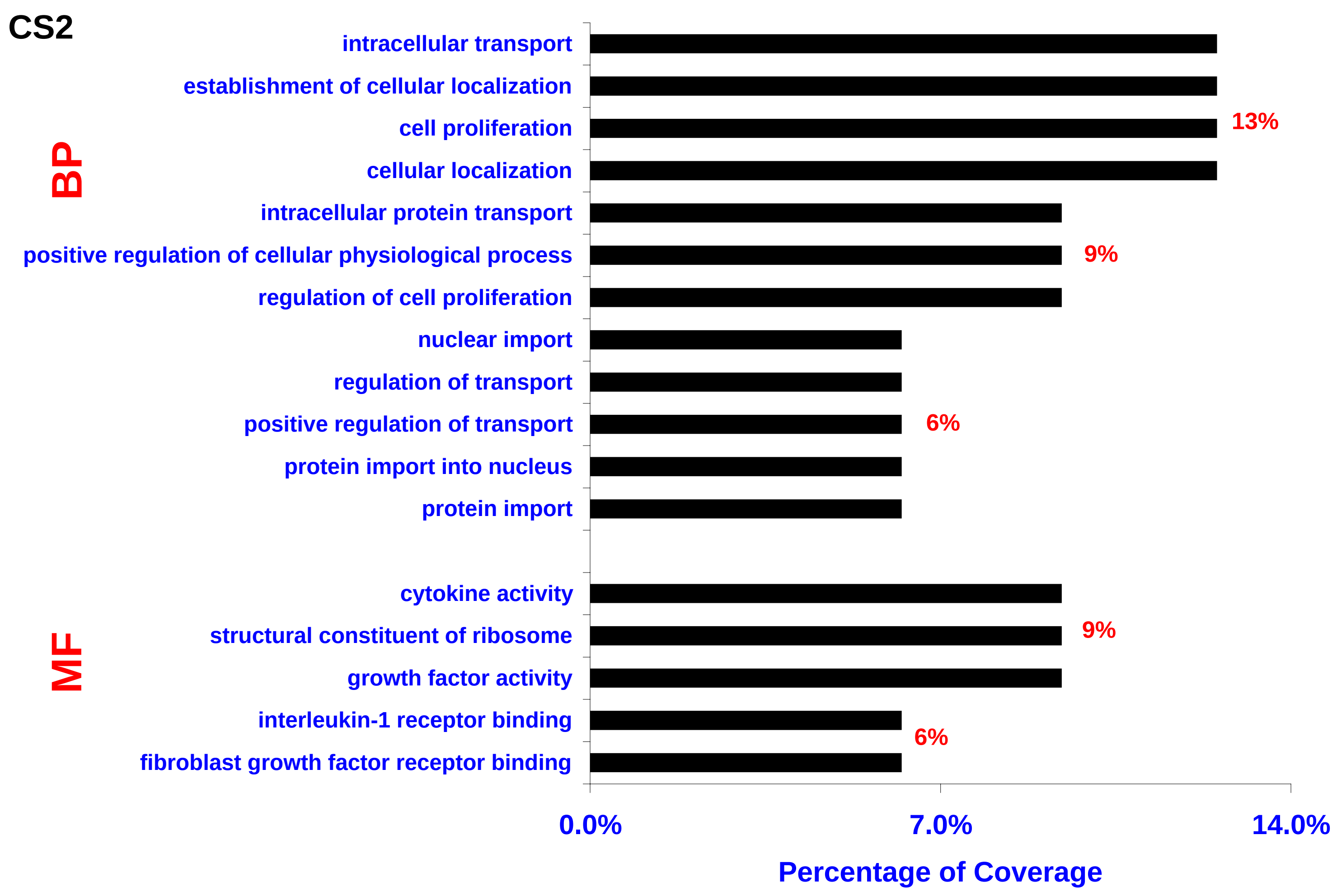
CS2
BP
intracellular transport
establishment of cellular localization
cell proliferation
cellular localization
intracellular protein transport
positive regulation of cellular physiological process
regulation of cell proliferation
nuclear import
regulation of transport
positive regulation of transport
protein import into nucleus
protein import
13%
9%
6%
MF
cytokine activity
structural constituent of ribosome
growth factor activity
interleukin-1 receptor binding
fibroblast growth factor receptor binding
9%
6%
0.0%
7.0%
14.0%
Percentage of Coverage

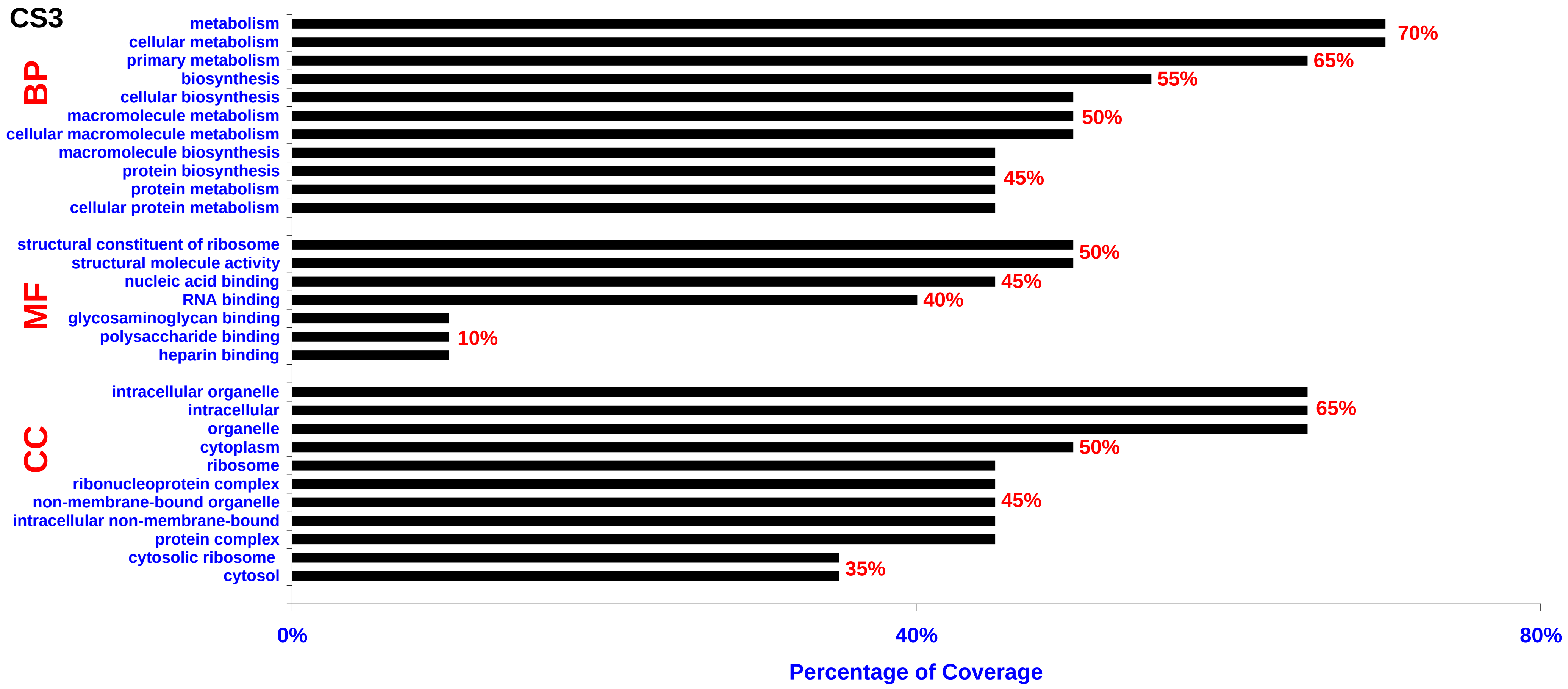
CS3
BP
MF
CC
metabolism
cellular metabolism
primary metabolism
biosynthesis
cellular biosynthesis
macromolecule metabolism
cellular macromolecule metabolism
macromolecule biosynthesis
protein biosynthesis
protein metabolism
cellular protein metabolism
structural constituent of ribosome
structural molecule activity
nucleic acid binding
RNA binding
glycosaminoglycan binding
polysaccharide binding
heparin binding
intracellular organelle
intracellular
organelle
cytoplasm
ribosome
ribonucleoprotein complex
non-membrane-bound organelle
intracellular non-membrane-bound
protein complex
cytosolic ribosome
cytosol
70%
65%
55%
50%
45%
50%
45%
40%
10%
65%
50%
45%
35%
0%
40%
80%
Percentage of Coverage

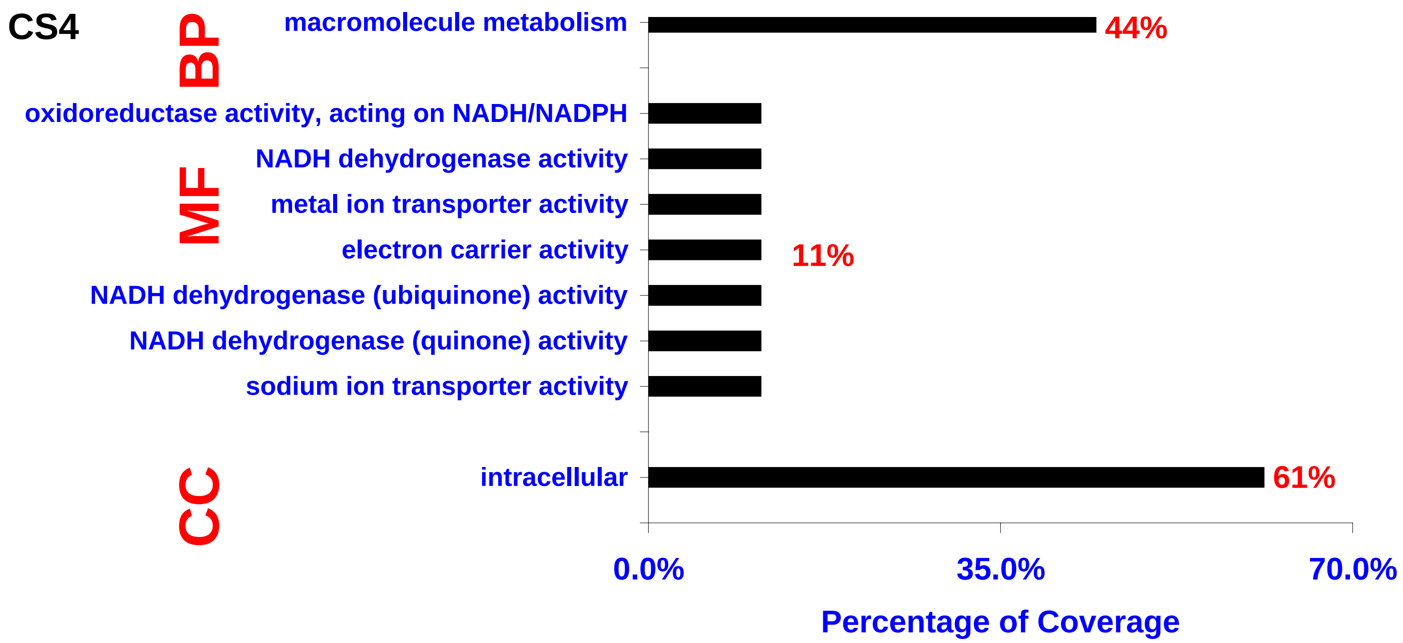
CS4
BP
macromolecule metabolism
44%
MF
oxidoreductase activity, acting on NADH/NADPH
NADH dehydrogenase activity
metal ion transporter activity
electron carrier activity
NADH dehydrogenase (ubiquinone) activity
NADH dehydrogenase (quinone) activity
sodium ion transporter activity
11%
CC
intracellular
61%
0.0%
35.0%
70.0%
Percentage of Coverage

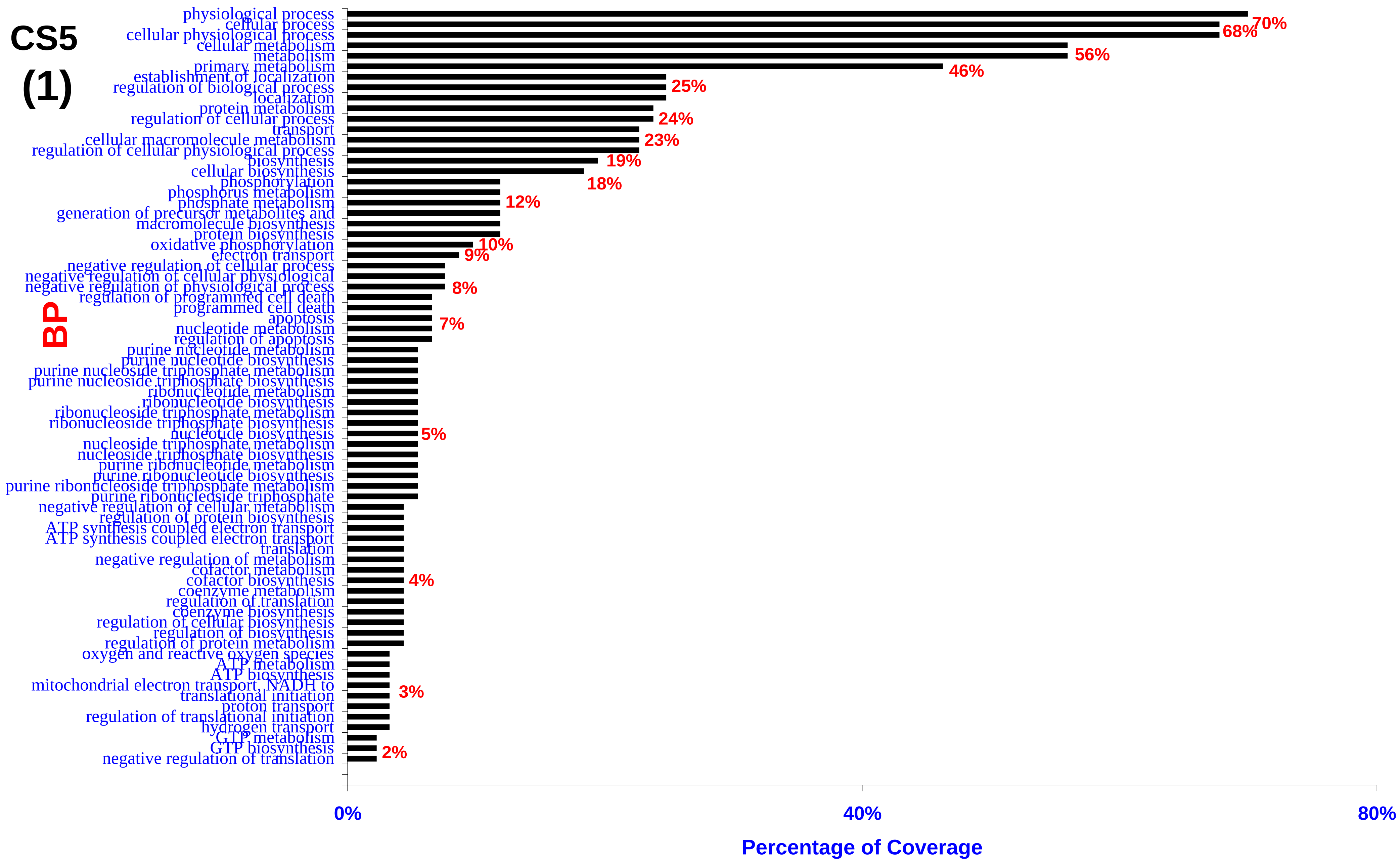
CS5
(1)
BP
physiological process
cellular process
cellular physiological process
cellular metabolism
metabolism
primary metabolism
establishment of localization
regulation of biological process
localization
protein metabolism
regulation of cellular process
transport
cellular macromolecule metabolism
regulation of cellular physiological process
biosynthesis
cellular biosynthesis
phosphorylation
phosphorus metabolism
phosphate metabolism
generation of precursor metabolites and
macromolecule biosynthesis
protein biosynthesis
oxidative phosphorylation
electron transport
negative regulation of cellular process
negative regulation of cellular physiological
negative regulation of physiological process
regulation of programmed cell death
programmed cell death
apoptosis
nucleotide metabolism
regulation of apoptosis
purine nucleotide metabolism
purine nucleotide biosynthesis
purine nucleoside triphosphate metabolism
purine nucleoside triphosphate biosynthesis
ribonucleotide metabolism
ribonucleotide biosynthesis
ribonucleoside triphosphate metabolism
ribonucleoside triphosphate biosynthesis
nucleotide biosynthesis
nucleoside triphosphate metabolism
nucleoside triphosphate biosynthesis
purine ribonucleotide metabolism
purine ribonucleotide biosynthesis
purine ribonucleoside triphosphate metabolism
purine ribonucleoside triphosphate
negative regulation of cellular metabolism
regulation of protein biosynthesis
ATP synthesis coupled electron transport
ATP synthesis coupled electron transport
translation
negative regulation of metabolism
cofactor metabolism
cofactor biosynthesis
coenzyme metabolism
regulation of translation
coenzyme biosynthesis
regulation of cellular biosynthesis
regulation of biosynthesis
regulation of protein metabolism
oxygen and reactive oxygen species
ATP metabolism
ATP biosynthesis
mitochondrial electron transport, NADH to
translational initiation
proton transport
regulation of translational initiation
hydrogen transport
GTP metabolism
GTP biosynthesis
negative regulation of translation
70%
68%
56%
46%
25%
24%
23%
19%
18%
12%
10%
9%
8%
7%
5%
4%
3%
2%
0%
40%
80%
Percentage of Coverage

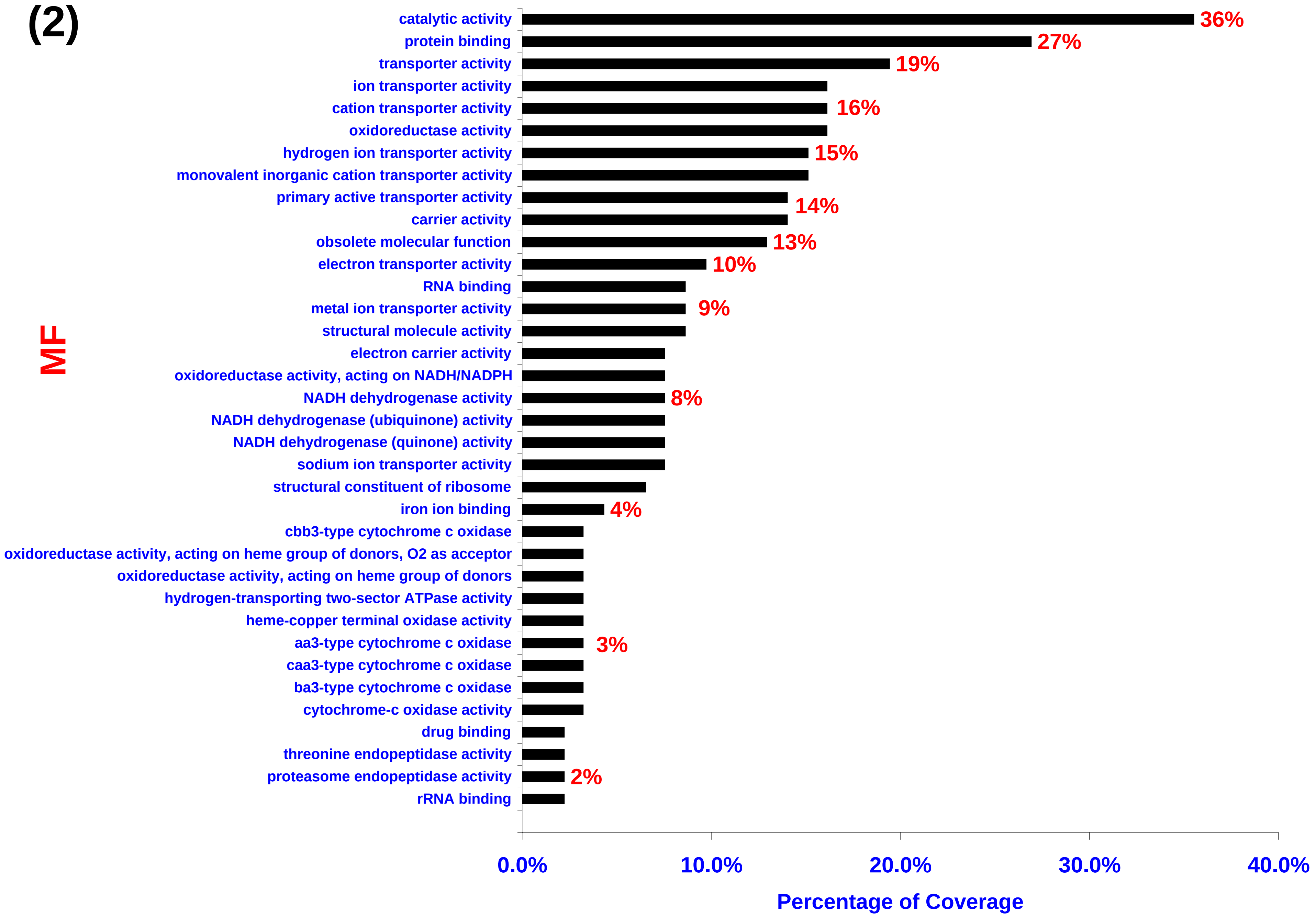
(2)
MF
catalytic activity
protein binding
transporter activity
ion transporter activity
cation transporter activity
oxidoreductase activity
hydrogen ion transporter activity
monovalent inorganic cation transporter activity
primary active transporter activity
carrier activity
obsolete molecular function
electron transporter activity
RNA binding
metal ion transporter activity
structural molecule activity
electron carrier activity
oxidoreductase activity, acting on NADH/NADPH
NADH dehydrogenase activity
NADH dehydrogenase (ubiquinone) activity
NADH dehydrogenase (quinone) activity
sodium ion transporter activity
structural constituent of ribosome
iron ion binding
cbb3-type cytochrome c oxidase
oxidoreductase activity, acting on heme group of donors, O2 as acceptor
oxidoreductase activity, acting on heme group of donors
hydrogen-transporting two-sector ATPase activity
heme-copper terminal oxidase activity
aa3-type cytochrome c oxidase
caa3-type cytochrome c oxidase
ba3-type cytochrome c oxidase
cytochrome-c oxidase activity
drug binding
threonine endopeptidase activity
proteasome endopeptidase activity
rRNA binding
36%
27%
19%
16%
15%
14%
13%
10%
9%
8%
4%
3%
2%
0.0%
10.0%
20.0%
30.0%
40.0%
Percentage of Coverage

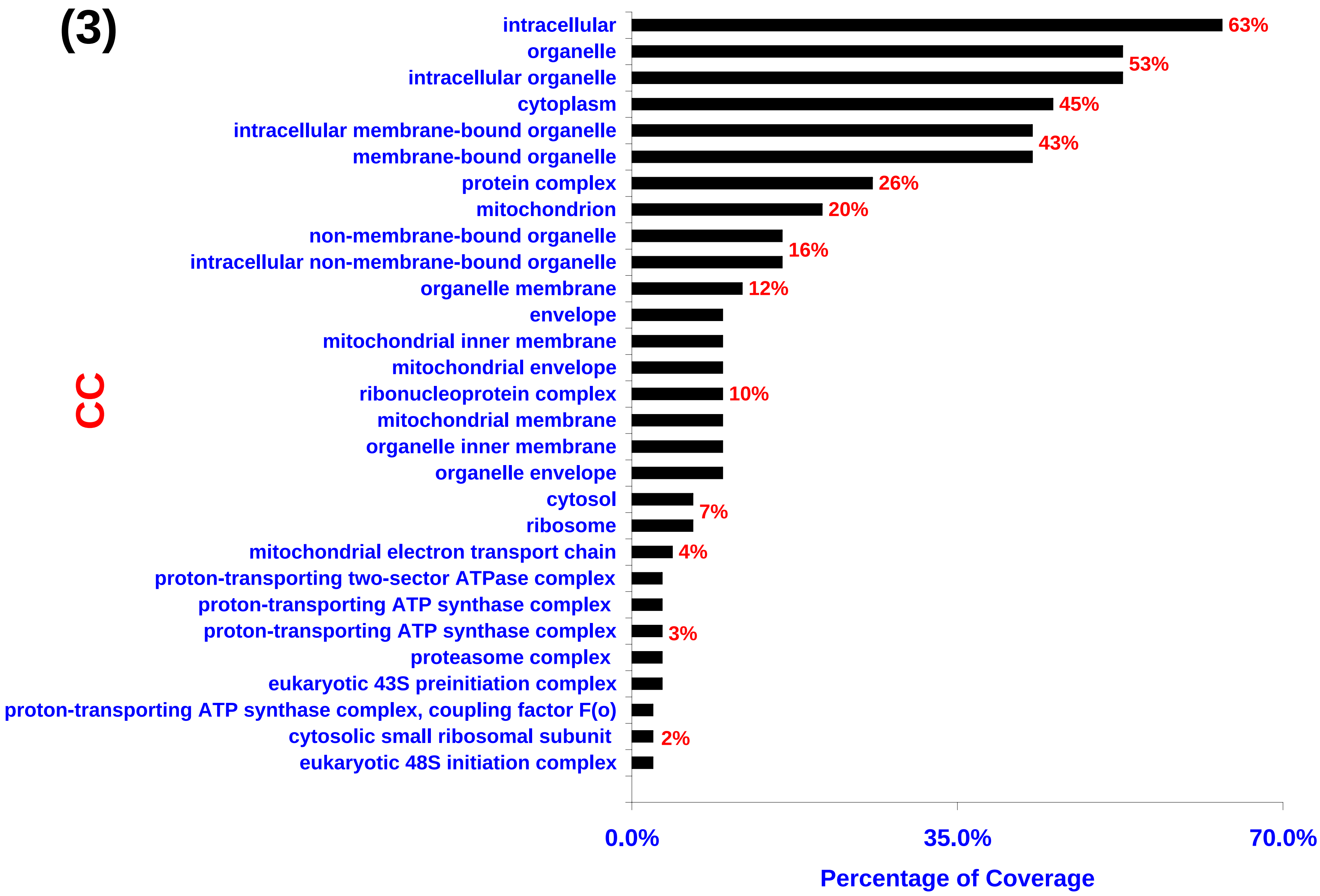
(3)
CC
intracellular
organelle
intracellular organelle
cytoplasm
intracellular membrane-bound organelle
membrane-bound organelle
protein complex
mitochondrion
non-membrane-bound organelle
intracellular non-membrane-bound organelle
organelle membrane
envelope
mitochondrial inner membrane
mitochondrial envelope
ribonucleoprotein complex
mitochondrial membrane
organelle inner membrane
organelle envelope
cytosol
ribosome
mitochondrial electron transport chain
proton-transporting two-sector ATPase complex
proton-transporting ATP synthase complex
proton-transporting ATP synthase complex
proteasome complex
eukaryotic 43S preinitiation complex
proton-transporting ATP synthase complex, coupling factor F(o)
cytosolic small ribosomal subunit
eukaryotic 48S initiation complex
63%
53%
45%
43%
26%
20%
16%
12%
10%
7%
4%
3%
2%
0.0%
35.0%
70.0%
Percentage of Coverage

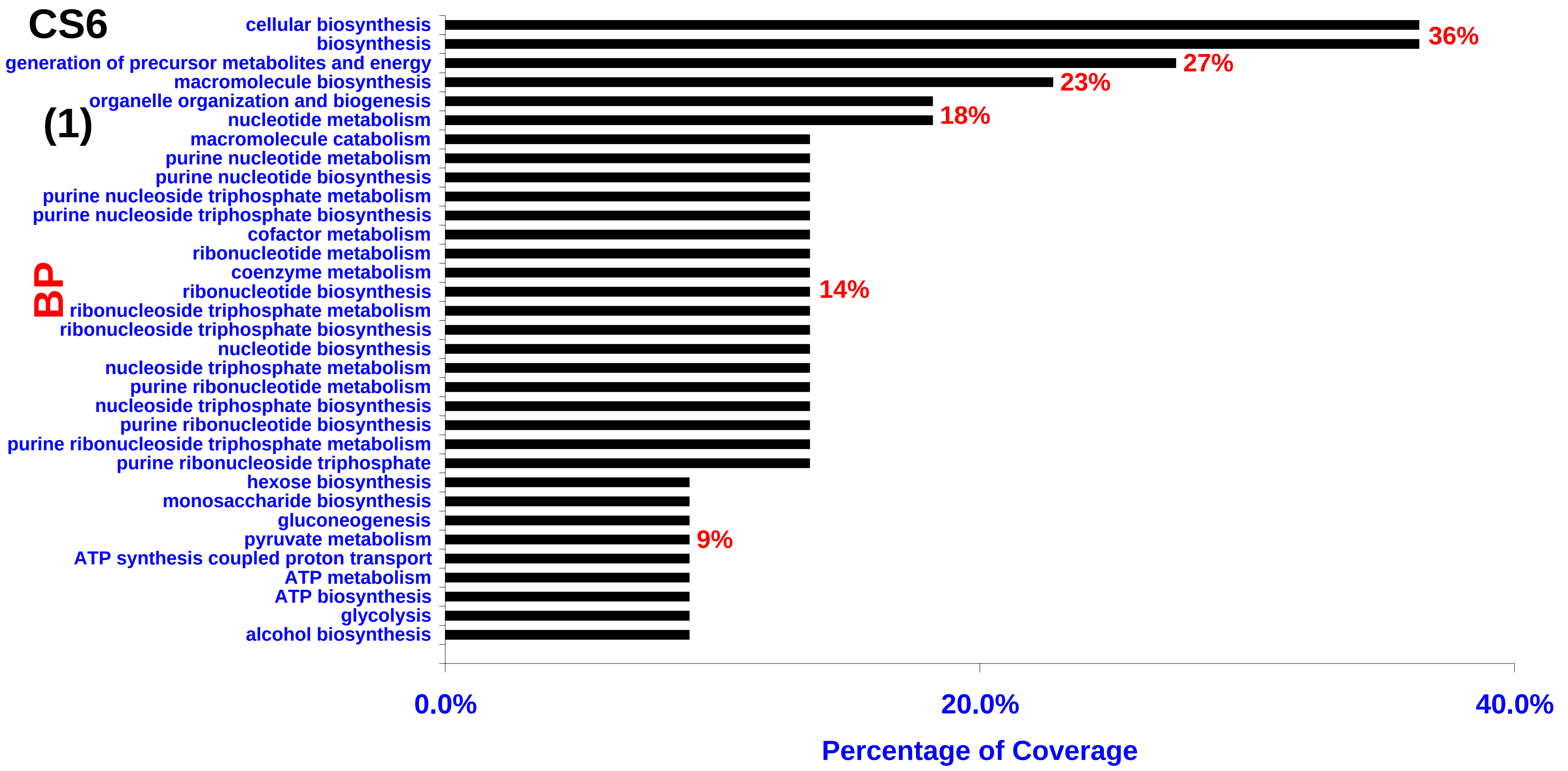
CS6
(1)
BP
cellular biosynthesis
biosynthesis
generation of precursor metabolites and energy
macromolecule biosynthesis
organelle organization and biogenesis
nucleotide metabolism
macromolecule catabolism
purine nucleotide metabolism
purine nucleotide biosynthesis
purine nucleoside triphosphate metabolism
purine nucleoside triphosphate biosynthesis
cofactor metabolism
ribonucleotide metabolism
coenzyme metabolism
ribonucleotide biosynthesis
ribonucleoside triphosphate metabolism
ribonucleoside triphosphate biosynthesis
nucleotide biosynthesis
nucleoside triphosphate metabolism
purine ribonucleotide metabolism
nucleoside triphosphate biosynthesis
purine ribonucleotide biosynthesis
purine ribonucleoside triphosphate metabolism
purine ribonucleoside triphosphate
hexose biosynthesis
monosaccharide biosynthesis
gluconeogenesis
pyruvate metabolism
ATP synthesis coupled proton transport
ATP metabolism
ATP biosynthesis
glycolysis
alcohol biosynthesis
36%
27%
23%
18%
14%
9%
0.0%
20.0%
40.0%
Percentage of Coverage

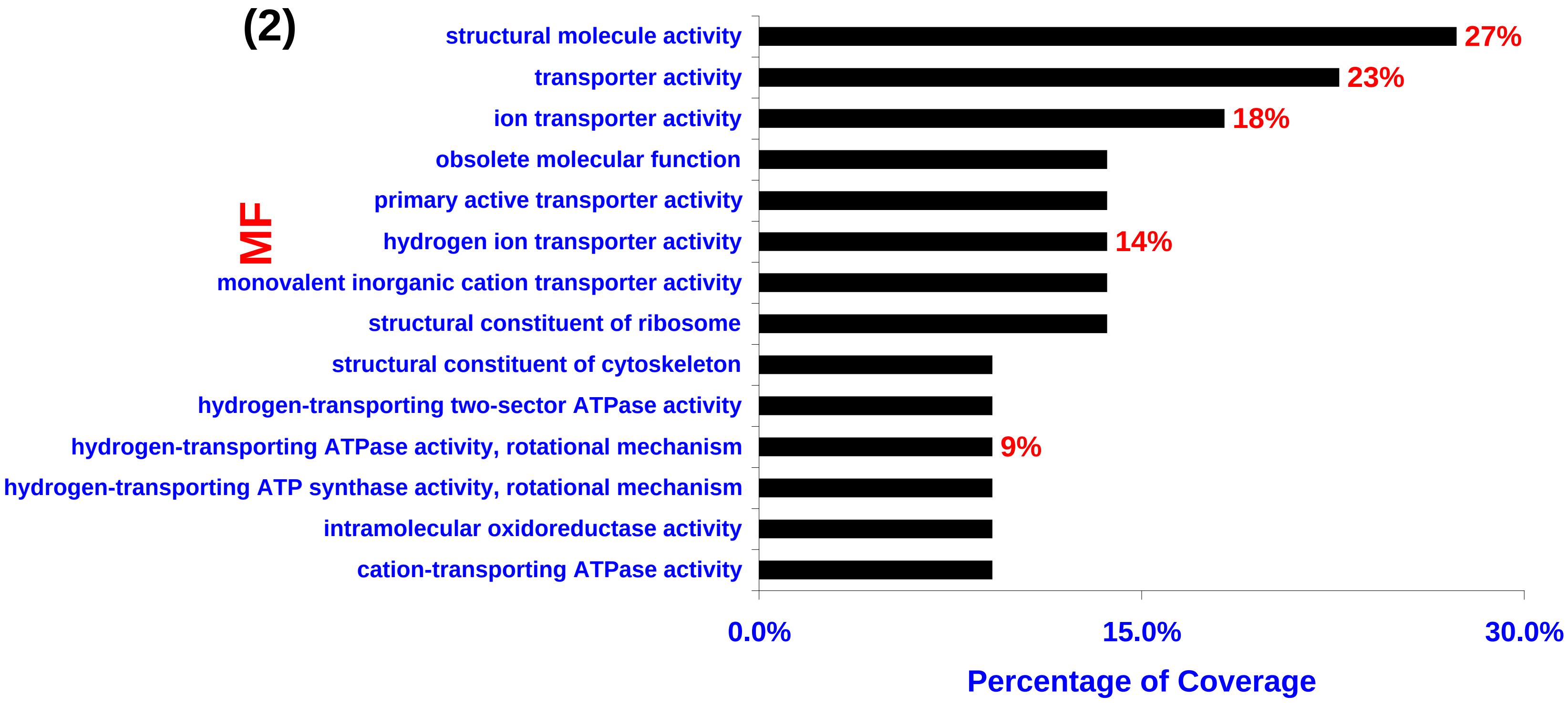
(2)
MF
structural molecule activity
transporter activity
ion transporter activity
obsolete molecular function
primary active transporter activity
hydrogen ion transporter activity
monovalent inorganic cation transporter activity
structural constituent of ribosome
structural constituent of cytoskeleton
hydrogen-transporting two-sector ATPase activity
hydrogen-transporting ATPase activity, rotational mechanism
hydrogen-transporting ATP synthase activity, rotational mechanism
intramolecular oxidoreductase activity
cation-transporting ATPase activity
27%
23%
18%
14%
9%
0.0%
15.0%
30.0%
Percentage of Coverage

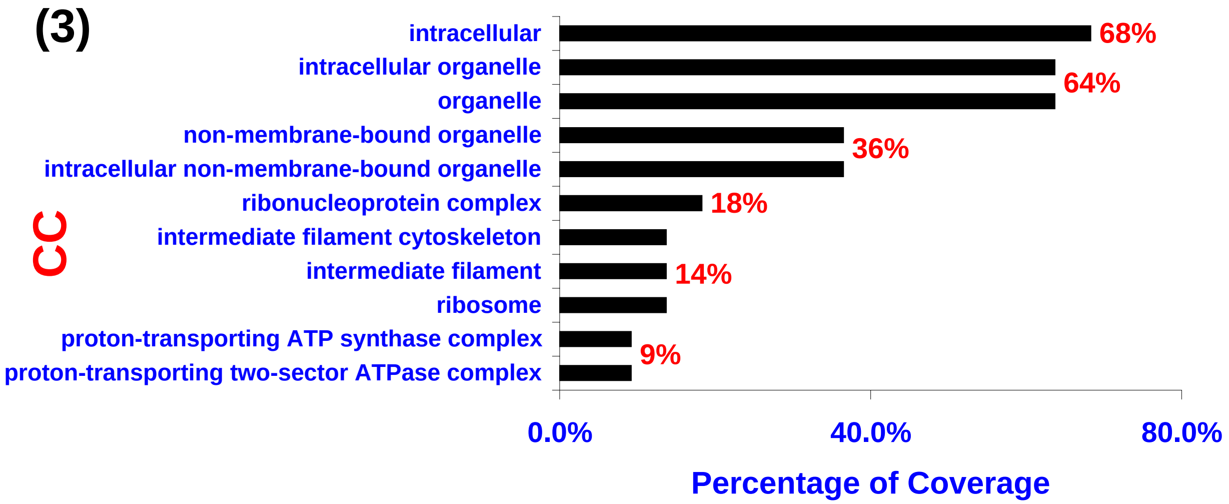
(3)
CC
intracellular
intracellular organelle
organelle
non-membrane-bound organelle
intracellular non-membrane-bound organelle
ribonucleoprotein complex
intermediate filament cytoskeleton
intermediate filament
ribosome
proton-transporting ATP synthase complex
proton-transporting two-sector ATPase complex
68%
64%
36%
18%
14%
9%
0.0%
40.0%
80.0%
Percentage of Coverage

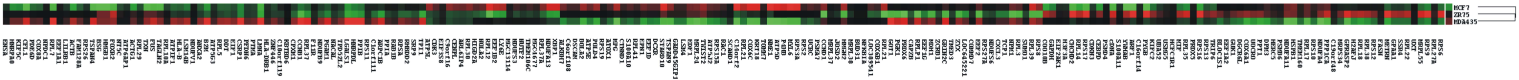

(i)

(ii)

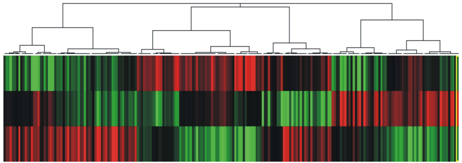

(iii)

Electron Transport Chain				Protein synthesis/processing							
Complex I											
	MCF-7	ZR75-1	MDA-MB-435		MCF-7	ZR75-1	MDA-MB-435		MCF-7	ZR75-1	MDA-MB-435
Total TPM	6329	8451	1693	Total TPM	91305	97050	34187	Total TPM	13143	7523	6409
NDUFV1				RPSA				EIF3S8			
NDUFS6				RPS9				EIF2AK1			
NDUFS3				RPS8				EIF1			
NDUFB9				RPS6				EEF2			
NDUFB1				RPS3A				EEF1G			
NDUFA4				RPS27A				EEF1D			
NDUFA3				RPS26				EEF1B2			
NDUFA13				RPS2				EEF1A2			
NDUFA11				RPS19				EEF1A1			
Complex IV				RPS17							
	5661	4489	1439	RPS16							
COX8A				RPS15A							
COX6C				RPS13							
COX6B1				RPS12							
COX6A1				RPS11							
Complex V				RPS10							
	11808	8203	1967	RPLP1							
ATP6AP1				RPL7							
ATP5L				RPL5							
ATP5J2				RPL41							
ATP5G3				RPL39							
ATP5E				RPL38							
ATP5D				RPL37A							
				RPL37							
Other genes (TPM)				RPL35							
NME1	1854	341	42	RPL30							
KRT18	6012	1672	212	RPL3							
STARD10	1486	898	21	RPL29							
FASN	418	836	169	RPL27A							
ATOX1	1019	991	148	RPL22							
NFKBIA	50	62	21	RPL21							
				RPL18							
Sugar Metabolism (Glycolysis)				RPL17							
Total TPM	1353	1053	2391	RPL14							
GAPDH				RPL12							
PGK1				RPL10A							
PGAM1											

Figure 1B

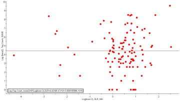

Figure 1C

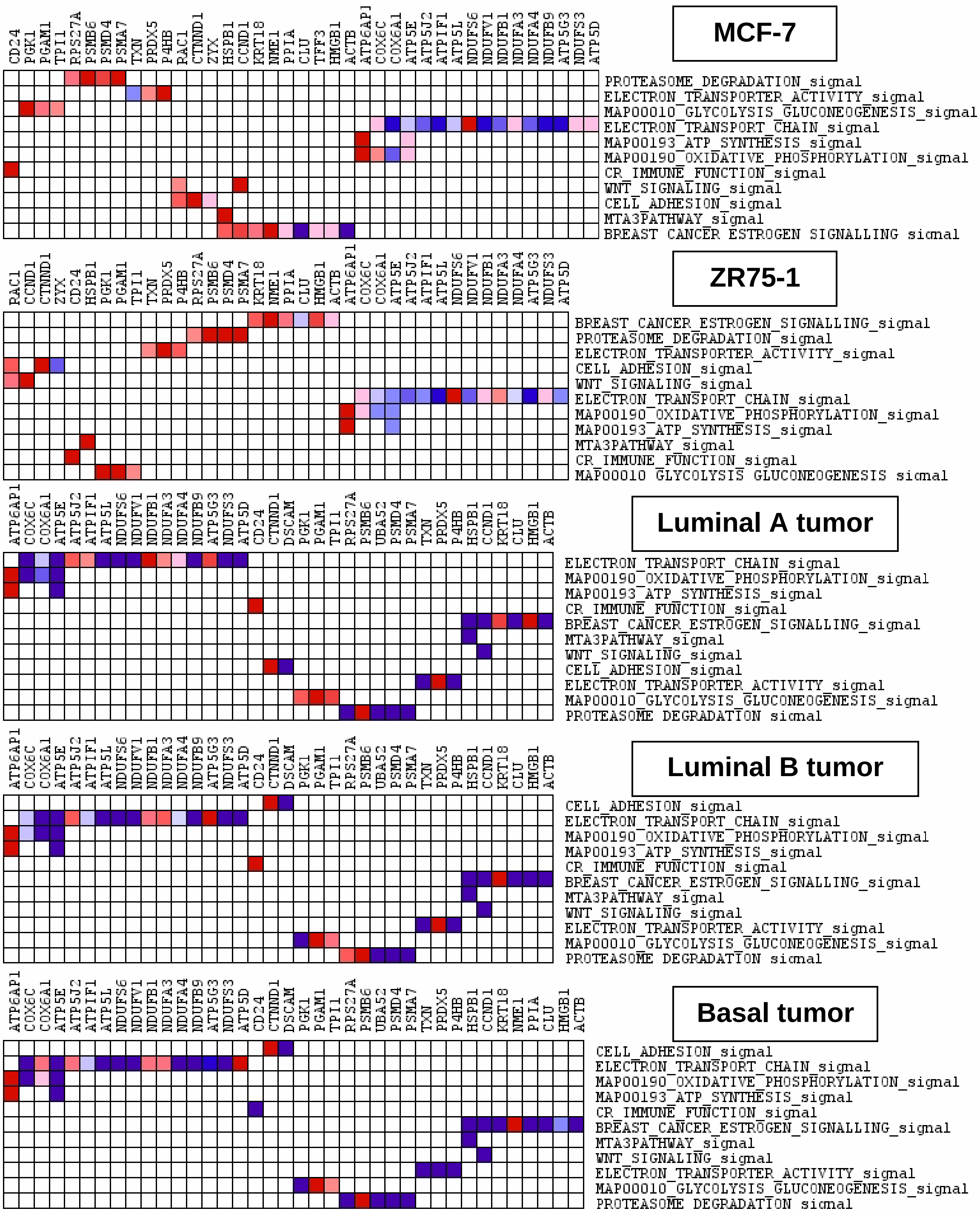

Figure 1D
